# Fouling Prevention in Polymeric Membranes by Radiation Induced Graft Copolymerization

**DOI:** 10.3390/polym14010197

**Published:** 2022-01-04

**Authors:** Muhammad Nidzhom Zainol Abidin, Mohamed Mahmoud Nasef, Takeshi Matsuura

**Affiliations:** 1 Chemical and Environmental Engineering Department, Malaysia-Japan International Institute of Technology, Universiti Teknologi Malaysia, Jalan Sultan Yahya Petra, Kuala Lumpur 54100, Malaysia; nidzhom@utm.my; 2 Center of Hydrogen Energy, Institute of Future Energy, Universiti Teknologi Malaysia, Jalan Sultan Yahya Petra, Kuala Lumpur 54100, Malaysia; 3 Department of Chemical and Biological Engineering, University of Ottawa, Ottawa, ON K1N 6N5, Canada; matsuura@eng.uottawa.ca

**Keywords:** organic fouling, pressure driven membrane processes, polymeric membranes, radiation induced graft copolymerization, biofilm formation, antifouling properties

## Abstract

The application of membrane processes in various fields has now undergone accelerated developments, despite the presence of some hurdles impacting the process efficiency. Fouling is arguably the main hindrance for a wider implementation of polymeric membranes, particularly in pressure-driven membrane processes, causing higher costs of energy, operation, and maintenance. Radiation induced graft copolymerization (RIGC) is a powerful versatile technique for covalently imparting selected chemical functionalities to membranes’ surfaces, providing a potential solution to fouling problems. This article aims to systematically review the progress in modifications of polymeric membranes by RIGC of polar monomers onto membranes using various low- and high-energy radiation sources (UV, plasma, γ-rays, and electron beam) for fouling prevention. The feasibility of the modification method with respect to physico-chemical and antifouling properties of the membrane is discussed. Furthermore, the major challenges to the modified membranes in terms of sustainability are outlined and the future research directions are also highlighted. It is expected that this review would attract the attention of membrane developers, users, researchers, and scientists to appreciate the merits of using RIGC for modifying polymeric membranes to mitigate the fouling issue, increase membrane lifespan, and enhance the membrane system efficiency.

## 1. Introduction

Membrane separation processes have received an ever growing interest in the past four decades, and the world has witnessed the remarkable rise in their large-scale applications in various fields, including food, water treatment, and medical and energy applications [1]. To date, membrane fouling is the major serious problem which is often encountered upon applying pressure driven membrane processes for solid/liquid separations such as microfiltration (MF), ultrafiltration (UF), nanofiltration (NF), and reverse osmosis (RO) [2]. It leads not only to a reduction in the flux and lifespan of the membrane that is coupled with an increase in the differential pressure and feed pressure, but also a reduction in the treated water quality, rise in the energy consumption and operation cost, and eventual deterrent of the widespread application of membrane technology [3]. Particularly, membrane processes such as water and wastewater treatment [4], desalination [5], dairy processing [6], fruit juice concentration [7], and whey protein concentration [8] are among those in which fouling typically poses a major drawback, impeding an efficient membrane performance.

Fouling is a complex phenomenon that occurs on membranes’ surfaces, or within their pores in liquid-based separations, because of the interactions between the foulants (e.g., bacteria, proteins, debris, and crystals) with the membranes’ rough surfaces, causing cake formation, organic adsorption, gel layer formation, inorganic precipitation, biological/microbial fouling, and full-to-partial pore blocking, all of which adversely affect the overall performance of the membrane systems [4,9]. The type and level of fouling depend on the fluid mechanics of the membrane system, the properties of the feed solution, and the characteristics of the membrane [10]. Thus, various methods have been used to reduce the membrane fouling by eliminating its promoting factors [7]. This includes pre-treatment of the feed with a disinfectant, backwash cleaning of the membrane, and optimization of the system operating conditions [11]. Other antifouling practices include modification of the membrane surface during its fabrication to endow the membrane with an intrinsic resistance to fouling. This is carried out by imparting hydrophilicity, roughness, and other characteristics to the membrane surface [3]. Membrane modifications can be performed by various methods including surface coating [12], blending, which introduces bulk modification [13], combining surface modification with blending [14], chemical treatment [15], interfacial polymerization [16], graft copolymerization [17], and incorporation of inorganic metal oxides additives such as titania [18], alumina [19], zirconia [20], and silica [21] in addition to nanoparticles, carbon nanotubes [22], graphene oxide [23], and immobilization of antimicrobial additives such as silver nanoparticles [24] occur during membrane fabrication.

**Figure 1:**
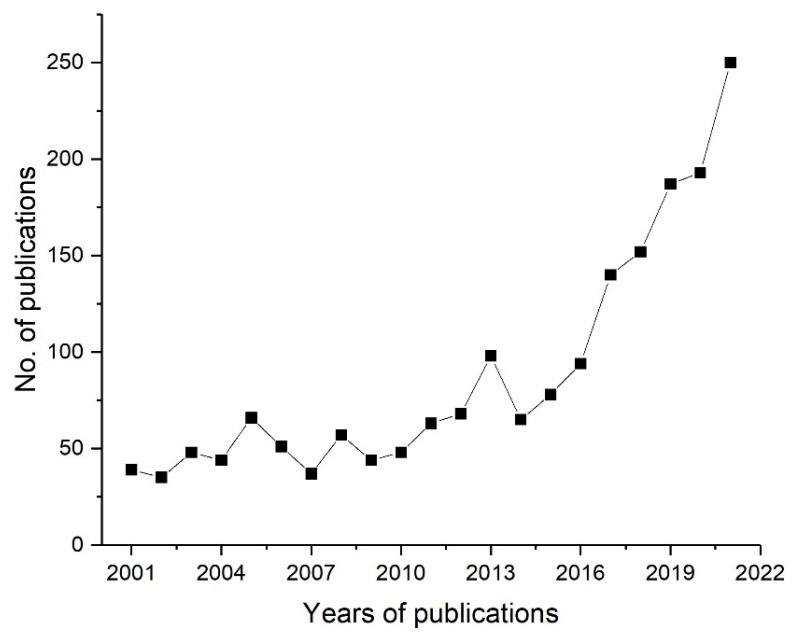
Number of publications on the use of RIGC for modification of polymeric membranes’ surfaces in various applications during the period of 2001–2021 (Science Direct, keyword search: radiation induced graft copolymerization, surface modification, polymeric membranes, antifouling, 5 October 2021).

Graft copolymerization, one of the techniques of interest that allow the covalent incorporation of the desired chemical functionality to the membrane surface, can be carried out using chemical grafting or radiation induced graft copolymerization (RIGC). The chemical grafting is a well-established method for the surface modification of membranes, but its challenges include leaving detrimental residues, difficulty in controlling the level of grafting, and posing environmental concerns because of hazardous chemical initiators and solvents. Alternatively, RIGC, which can be carried out by low-energy (UV and plasma) and high-energy (γ-rays and electron beam (EB)) radiation, is a promising method that allows modifications of the membrane surfaces by covalent immobilization of selective antifouling moieties, with controllable levels of grafting using less hazardous chemicals [25]. Nevertheless, application of this method has not received sufficient attention, despite its merits. The growing number of publications on the use of RIGC for polymeric membrane surface modifications in various applications during the past 20 years is shown in Figure 1. As can be seen, the number of publications is increasing exponentially, which suggests the growing interest in using the RIGC method to address the long-standing issue of membrane fouling and realize its potential.

Immense progressive research efforts have been made to understand the various aspects of fouling, and this progress was captured in several review articles and book chapters. This includes reviews on the fouling mechanism and key strategies for overcoming it [4,26], membrane antifouling coatings against biomolecules and protein [27,28,29,30], fouling in water treatment processes (e.g., desalination and RO) [3,4,9,31], and practiced as well as emerging eco-friendly technologies for fouling control [32]. On the other hand, few articles reviewed the progress in the antifouling modifications of the widely used membrane polymers such as poly(vinylidene fluoride) (PVDF) [33,34], the antifouling membrane surface construction using various chemistries [35], and the real-time fouling monitoring techniques [36]. However, there is a lack of information on the aspects of RIGCs as fouling mitigation methods working through membrane surface modifications with functional groups. Moreover, a review dealing with RIGC for modification of membranes using different radiation sources and the progress taking place to impart covalently attached antifouling properties is rather scarce [37].

The objective of this article is to provide a comprehensive review on the development of various methods used to modify the surface of polymeric membranes by RIGC, initiated with high- and low-energy radiation sources to reduce or prevent the fouling in membrane processes operating based on various separation driving forces. The scope of the article covers an overview of the pressure driven membranes processes. Particularly, it covers the mechanism of fouling and the factors that contribute to the membrane fouling. The fundamentals of RIGC for modification of the membranes’ surfaces to endow hydrophilic, ionic, and antifouling properties are briefly reviewed, followed by a discussion on the various types of radiation sources applied for membrane surface modifications. The challenges hampering the widespread application of RIGC in the membrane antifouling treatments and the future research directions to overcome them are also discussed.

## 2. Membrane Processes Based on Various Separation Driving Forces

Membrane processes are classified based on their operational driving forces, which further depend on their separation mechanisms, such as sieving [38], solution-diffusion [39], adsorption [40], and electrochemical effects [41]. Thus, the driving forces include the gradients of pressure [42,43,44,45], potential [46,47,48], and concentration [49,50] across the membrane. Pressure driven membrane processes include MF [51], UF [52], NF [53], and RO [54]. The main factors categorizing these membrane processes are pore size of the membrane and the magnitude of the applied transmembrane pressure (TMP). Generally, the pore size of the pressure driven membranes decreases in the order from MF to RO, while the operational TMP value increases from MF to RO [55].

**Figure 2:**
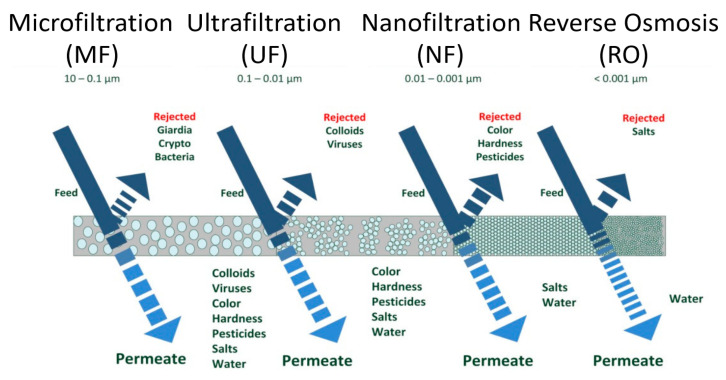
Classification of pressure driven membrane processes for water treatment technologies. Reprinted from [60], published by SDEWES Centre.

MF and UF are the most crucial membrane processes for various applications due to their economic operation, availability of the membranes with higher overall membrane flux, cheaper process cost, and lower fouling degree. Among the applications that use MF and UF are cell harvesting and sterile solution production. They are also used for the membrane bioreactors (MBR) and in the dairy products’ industry. On the other hand, the RO process works against the chemical potential difference, namely osmotic pressure [56], and hence the TMP applied in RO is normally much higher than other pressure driven membrane processes [55]. NF has specific applications such as water purification [57], brackish water desalination [58], and water softening [59]. RO and NF are typically evaluated by the permeability of water and the rejection of mono- and di-valent ions from salts such as NaCl and MgSO_4_, respectively. In short, each of these processes has its own meritorious practicality in serving different applications. A summary of the basic operating principles of the pressure driven membrane processes is illustrated in Figure 2.

Potential driven membrane process is an alternative process that uses potential difference or stored energy as the main driving force [61]. This potential energy is suitable for promoting membrane processes aiming to transport individual species between two phases by means of electrochemical effect or osmosis. Examples of electrical potential membrane (electromembrane) processes are electrodialysis (ED) and electrodialysis reversal (EDR), in which ions are transferred through cation exchange membrane (CEM) and anion exchange membranes (AEM) [62]. The applications of ED include heavy metal removal, brackish water demineralization, the chlor-alkali industry, and energy storage [2]. In EDR, the voltage applied to the electrodes is reversed intermittently, allowing the flow of the cations and anions to be reversed and enabling the removal of scale and foulants deposited on the membranes’ surfaces [63].

Chemical potential as the main operational driving force gave birth to various membrane processes, such as forward osmosis (FO), which is a process of transporting water across a semi-permeable membrane from a higher water chemical potential region (low osmotic pressure) to a lower water chemical potential region (high osmotic pressure) [46]. FO is usually used as a pre-treatment for wastewater. Pressure retarded osmosis (PRO), which is the inverse process of RO, is considered an excellent technology utilizing salinity gradient to produce electricity [64]. PRO uses the osmotic pressure of salt water to mix purified water with a saline water and, naturally, generates pressure energy that can be converted into mechanical/electrical energy. Reverse electrodialysis (RED), which is a newly emerging electrochemical driven membrane process, also utilizes the salinity gradient to produce electricity [65]. RED cells directly generate electricity from the difference in the salinities of the feed waters, commonly fresh water and the saline water [66]. The chemical energy difference of the two solutions separated by the ion exchange membranes generates potential at the cell electrodes [67].

Concentration driven membrane processes involve the transport (separation) of targeted components (solutes) from the higher concentration side to the lower concentration side via diffusion until the equilibrium is achieved. The most common concentration driven membrane process is dialysis, typically applied in hemodialysis systems. Hemodialysis involves passing the human blood through a dialyzer to remove wastes such as urea and excess water [68]. Inside the dialyzer, as blood moves in the membrane module through the lumen side, the outer side of the membrane comes in contact with dialysis water [69], hence generating a concentration difference for the diffusion to happen.

## 3. Membrane Fouling

**Figure 3:**
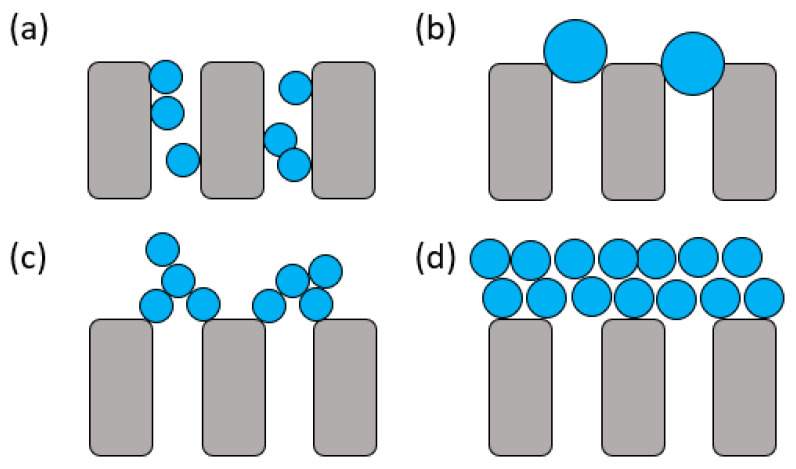
Various mechanisms for membrane fouling: (**a**) standard blocking, (**b**) complete blocking, (**c**) intermediate blocking, and (**d**) cake formation.

Membrane fouling is a phenomenon of gradual decline in the permeation rate during the membrane process caused by the deposition of the retained feed components, such as proteins, over the membrane surface, or the adsorption of solutes internally, inside the membrane pores [52]. Fouling takes place through four common mechanisms, as shown in Figure 3; each is caused by a different potential scenario [70]. Standard blocking involves particle adsorption or deposition within the pores (Figure 3a). The accumulation of these feed components in the membrane pores increases the particle size of foulants, resulting in a pore clogging or a complete blocking (Figure 3b). This phenomenon is most common in MF and UF membranes. Meanwhile, the intermediate blocking happens when the feed components are accumulated layer-by-layer over the membrane surface (Figure 3c). The accumulation of the foulant layers, in addition to the concentration polarization of the feed components over the membrane surface, would finally lead to cake formation (Figure 3d). In practice, the relatively denser and more compact semi-permeable membranes, such as those used in NF and RO, experience cake formation on their surfaces.

### 3.1. Membrane Properties Contributing to Fouling

The relationship between the membrane surface’s properties and fouling has been ever in the focus of investigations since the beginning of membranes’ application for solid/liquid separations. The rate and severity of the membrane fouling are found to be greatly dependent upon the parameters pertaining to both feed water qualities and membrane surface properties [71,72]. Particularly, the membrane surface properties are known to determine the way the membrane interacts with foulants [10,73].

#### 3.1.1. Surface Hydrophilicity

Fouling is more severe in the hydrophobic membranes and is caused by the hydrophobic interactions between solutes, microbial cells, and the membrane materials [74]. Surface hydrophilicity of a membrane is determined by the contact angle measurement [75]. Most of the commercial pressure driven membranes are made of hydrophobic polymers such as PVDF, polyethersulfone (PES), polysulfone (PS), poly (ether ether ketone) (PEEK), polytetrafluoroethylene (PTFE), polypropylene (PP), polyacrylonitrile (PAN), polyamide (PA), and polyethylene (PE). Enhancing surface hydrophilicity can be achieved by increasing the density of the hydrophilic groups, such as hydroxyl and amine, at the membrane surface [76]. Hydrophilic membranes have a thin layer of bound water on their surfaces that helps to prevent or reduce the foulant adsorption or adhesion at the membrane surface.

#### 3.1.2. Surface Roughness

There is a strong correlation between the surface roughness and membrane fouling behavior [10,77]. Commonly, the flux decreases as the surface roughness of the membrane increases [38]. The ridge–valley structure, that can be visualized under atomic force microscopy (AFM), favors foulant accumulation at the surface, and a greater roughness increases the total surface area to which foulants can be attached [78]. Therefore, the membranes with rougher surfaces are more prone to foulant attachment, resulting in faster fouling rates. However, it has been also reported that the increase in the surface roughness leads to an increase in the flux, and this is attributed to the increased area available for the membrane transport of liquid [30].

#### 3.1.3. Surface Charge

Membrane surface charge, quantified by the zeta potential measurement [40], is highly critical for reducing membrane fouling by charged foulants. Negative surface charges of a membrane are commonly formed by imparting sulfonic [79] and carboxylic acid [80] groups, which dissociate in the feed solution, whereas positive charge is caused by the presence of protonated amine [81] and quaternary ammonium salt groups [82]. Electrostatic repulsion between the solute and the membrane prevents the solute deposition on the membrane and, thus, reduces the fouling of charged organic compounds with similar charges to the membrane surface [83]. For instance, negatively charged membranes are used for separation of negatively charged proteins, since they exhibit electrochemical repulsion against each other [84], although, in some cases, their interaction with opposite charged ions can form precipitates [85], accelerating the membrane fouling.

### 3.2. Fouling Classifications

Membrane fouling is generally classified according to the type of the foulant. The main classes of fouling include: (i) colloidal fouling [86], (ii) organic fouling [87], (iii) inorganic scaling [88], and (iv) biofouling [89]. Table 1 shows the fouling profiles of different membrane processes. In the case of MF, UF, NF, and RO, the fouling severity increases with the decreasing membrane pore size. Meanwhile, the insoluble salts found in the brackish water and inorganic colloids found in the river water contribute to the fouling for ED/EDR and RED, respectively. In FO and PRO, the presence of micropollutants in the feed and the higher concentration of salts in the draw solution may cause deposition and accumulation of the foulants on both sides of the membrane surface. Of all membrane processes, a pressure driven process is highly exposed to fouling due to the sieving effect, whereby the rejected molecules are pressurized against the membrane surface [90]. Another application with a high tendency for fouling is hemodialysis, whereby the rapidly adsorbed proteins onto the surface of the membrane can cause higher platelet adhesion, fast blood coagulation, and aggregation.

## 4. Strategies for Fouling Prevention

Several strategies have been adopted to reduce the membrane fouling through addressing its route causes. These antifouling strategies include surface modification [7] and bulk modification [91] of the membranes. The methods to prepare the antifouling membranes by introducing functionalities such as hydrophilic moieties or charged groups can be mainly classified into surface coating, blending, and grafting. The graft copolymerization method enables covalent attachment of the functional groups and thus imparts desired properties to the membranes. Obviously, the last method is superior regarding resistance to the functional group leaching and fouling. More details on the strategies to reduce the membrane fouling, such as dip coating, layer by layer assembly, blending, and interfacial polymerization, can be found elsewhere [92,93,94,95].

### 4.1. Graft Copolymerization

**Figure 4:**
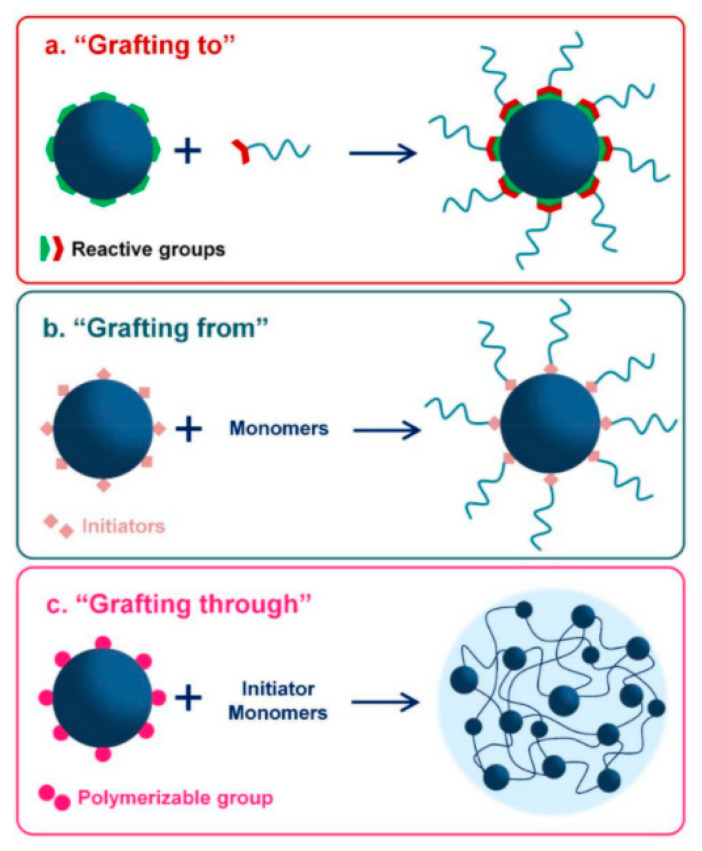
Schematic representation of methods for preparation of graft copolymers. Reprinted from [98].

Graft copolymerization is a reaction in which side chain grafts, originated from one or more vinyl monomers, are covalently attached to a linear polymer backbone leading to formation of graft copolymer products, that have new characteristics, originated from two or more parent polymers. The sequence of the monomer units varies depending on the distinct reactivity of the monomers during the polymerization process. Three approaches are usually used for the preparation of graft copolymers. “Grafting to” includes the reaction of functional groups on two different polymers, as schematized in Figure 4. “Grafting from” involves polymerization reaction between a polymer with functional groups (macro-initiator) with monomers. “Grafting through” contains polymerization of macromonomer(s) [96,97].

**Figure 5:**
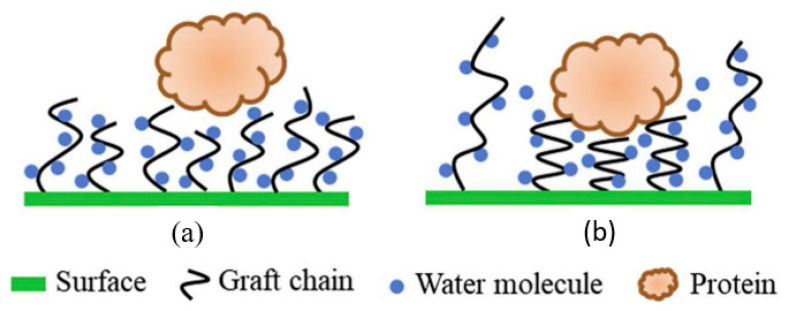
Illustrations of antifouling approaches with copolymer grafted membranes: (**a**) steric repulsion preventing direct protein adsorption and (**b**) protein compressing the polymer brush. Reprinted from [99] with permission from Elsevier.

In the “grafting from” approach, that has been broadly carried out in the modification of polymeric membranes, the polymer grows from the main polymer backbone by conventional polymerization. The process involves formation of the paramagnetic types (radicals or charged intermediates) on the main polymer backbone. These active polymers react with the monomer molecules and initiate a polymerization reaction. In terms of fouling prevention, the large chain density of the grafted polymer closes the gap between polymer chains, making such gaps much smaller than the size of the protein. This causes difficulty for the protein molecules’ adsorption on the membrane surface through the voids, as schematized in Figure 5a. As the protein approaches the membrane surface, the longer grafting chains, due to their high grafting density, increase the degree of compression of polymer brushes to impede the protein molecules (Figure 5b) [99]. This means that polymer brushes with long graft chains may have strong steric repulsion to proteins, which can help to improve the membrane’s antifouling performance.

Graft copolymerization for membrane modifications can be initiated by various methods, such as surface chemical treatment [100], chemically induced graft copolymerization [33], and RIGC, that can be initiated by high energy radiation such as γ-rays and EB [101] or low energy radiation such as UV and plasma [83]. However, chemical grafting is marred by environmental concerns over the post reaction residues containing hazardous chemical initiators and solvents and leading to production of a lot of wastewater, although it was commonly applied as a finishing technique for sheets and fabrics (stain repellence, flame retardance, dyeing, and antibacterial treatments) [25]. Moreover, the chemical grafting method is associated with the difficulty in shaping functionalized polymers into uniform and pin-hole free membranes. Of all membrane modification methods, RIGC is an interesting method that allows controlled modifications of the membranes by covalent immobilization of the antifouling agents to desired levels, without leaving detrimental residues and avoiding membrane shaping problems [101,102,103].

### 4.2. Radiation Induced Graft Copolymerization

RIGC is a facile and convenient method for selectively and covalently imparting new properties originated from polar monomers into the polymeric substrates without altering their inherent properties and by using a variety of radiation sources. This method provides desired control over the type and level of grafted moiety, and the grafting yield as a function of grafting parameters [104]. Moreover, it helps maintain the purity of the product, which is free of detrimental residues and thus exerts lower environmental impact and provides an eco-friendly antifouling approach [105]. Thus, RIGC has been applied for the modification of surfaces for controlling the biofilm formation, bacterial adhesion, and growth in various occasions [106,107,108].

#### 4.2.1. Low Energy Radiation

RIGC can be carried out by low energy radiation such as UV light, a technique that can be renamed as photo induced grafting. When UV light falls on a polymer, active species (radicals, cations, or, rarely, anions) are formed. Among formed species, radicals are the most active species to become involved in radical polymerization reactions. RIGC using UV treatment has been widely used and accepted for modification of non-photoactive materials with vinyl monomers in the presence of initiating agents or photo-initiators, such as benzophenone (BP) and 1-hydroxycyclohexyl phenyl ketone. Several types of photo-initiators have been used, and details of their classification can be found elsewhere [109]. The reaction of a photo-initiator with the base membrane polymer under UV irradiation should generate initiating radical sites at the membrane surface. A grafting reaction is commonly carried out using the immersion technique, where UV radiation is used in the presence of vinyl monomer diluted with water or methanol. The selection of an appropriate wavelength is a critical factor in this method, which is suitable for imparting new ionic characters to surfaces of polymeric membranes [110]. However, UV grafting is slow, yields low grafting levels, and needs a photo-initiator. Moreover, while it is rather effective for modification of small scale samples, it is not practical for industrial large-scale applications [110].

Plasma induced graft copolymerization is an attractive means for the development of antimicrobial and antifouling coatings using various plasma systems [111,112]. Plasma treatment provides a unique method for grafting on the surface of polymer substrates through inelastic collisions with active species in the presence of monomer precursors (fluorocarbons, hydrocarbons, and silicon) or polymerizable gases (e.g., NH_3_, N_2_, O_2_, CO_2_, or H_2_O), leading to energy gain, activation, and grafting initiation [113]. The reaction proceeds by either a radical generated on polymer surfaces followed by contact with monomers or direct grafting of polymer surfaces with activated monomers. The level of grafting depends on the plasma treatment conditions, including monomer reactivity, flow rate, system pressure, discharge power, frequency of the excitation signal, and the temperature of the substrate [114]. Particularly, low pressure plasma (LPP) and atmospheric pressure plasma (APP) treatments have been employed for the polymer surface treatment by RIGC [115]. The grafting yield and length of grafted chains can be not only controlled by plasma parameters such as power, pressure, treatment time, and sample disposition, but also by polymerization conditions, including monomer concentration, type of solvent, and grafting time [115]. The use of plasma techniques provides several advantages, including being green and suitable for providing uniform surface modifications of polymeric substrates without bulk damage. Thus, it is a cost effective and efficient way to impart desired interfacial properties to textiles [116] and membrane surfaces [117]. More details on the application of plasma induced grafting for imparting functional properties and antimicrobial coating to various polymer surfaces were discussed in a previous analysis [118].

#### 4.2.2. High Energy Radiation

**Figure 6:**
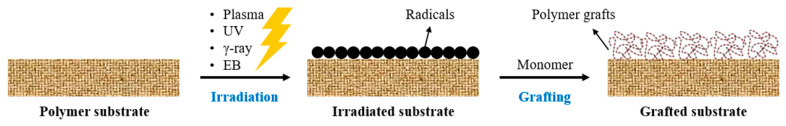
Schematic diagram of strategies for functionalization of polymer surfaces by RIGC using different radiation sources.

RIGC is commonly carried out by high energy/ionizing radiation such as γ-rays from Co-60 and accelerated electrons from EB accelerators. Ionizing radiation from less used radiation sources such as swift heavy ions was also used to perform RIGC reactions, but is commonly applied for track etching and formation of regular (cylindrical or conical) nanochannels in the polymeric membranes [119,120]. The interaction of ionizing radiation with polymer substrates leads to the formation of active cites or free radicals via H atom abstraction from hydrocarbon polymers (e.g., PP), or F atom in case of fluoropolymers (e.g., PTFE), leading to formation of radicals on the polymer backbone and initiating graft copolymerization upon contacting monomer molecules [101]. Two main methods can be used for graft copolymerization: (i) simultaneous irradiation, where both a polymer substrate and a monomer are usually exposed to γ-rays under controlled atmosphere, in the presence of a small amount of an inhibitor (Fe^2+^ or Cu^2+^) to minimize the homopolymerization and (ii) pre-irradiation, in which the substrate is independently irradiated and then brought into contact with the monomer under either a vacuum/inert or oxygen atmosphere. The resulting trapped radicals, or hydroperoxyl radicals, can be used for the reaction initiation by the thermal decomposition (i.e., heating grafting mixture) in the presence of a monomer. γ-rays and EB induced grafting can achieve modification beyond polymer surfaces and have been widely investigated for the preparation of various ion exchange and chelating polymers [118]. Simultaneous irradiation with γ-rays is rather slow, but is more suitable for bulk modification of radiation-sensitive polymer substrates. On the other hand, EB is a fast irradiation source for surface grafting and is a more convenient pre-irradiation method for large scale applications. The penetration depth of EB depends on the electron acceleration energy and the density of the substrate [101]. Various accelerators of different acceleration energy, varying from low energy (0.1–0.3 MeV) to medium energy (0.5–5 MeV) and high energy (5–10 MeV) types, are available for surface curing and polymer processing [121]. Figure 6 shows a schematic diagram of strategies for the functionalization of polymer surfaces by RIGC using different radiation sources. The level of desired polymer modification can be easily optimized by controlling the grafting conditions, including the radiation source, monomer concentration, absorbed dose, dose rate, temperature, and time. More elaborations on the merits and demerits of common methods used for modification of the polymeric membranes by RIGC are presented in Table 2. In general, membrane modification using physical or non-covalent methods has many drawbacks, such as particle leaching and a non-uniform surface, which bring inconsistency to the membrane performance results. On the contrary, the covalent chemical modification methods are more effective in providing stable modifications, but chemical initiators must be avoided to eliminate the hazardous environmental impact.

## 5. Progress in Application of RIGC for Fouling Prevention

RIGC has been proposed as a very convenient and promising method for imparting antifouling characteristics to the polymeric membranes, and thus it became the subject of many investigations. The antifouling characteristics are introduced by incorporation of functional polymers that originated from various monomers such as acrylic acid (AA), acrylamide (AAm), 2-acrylamidoglycolic acid (AAG), 2-acrylamido-2-methyl-1-propanesulfonic acid (AMPS), N-(3-tert-butyl-2-hydroxy-5-methylbenzyl) acrylamide (BHMBA), 2-(dimethylamino) ethyl acrylate (DMAEA), 2-(dimethylamino) ethyl methacrylate (DMAEMA), ethylene diamine (EDA), ethylene glycol dimethacrylate (EDMA), ethylene glycol dimethyl ether (EGDME), D-gluconamidoethyl methacrylate (GAMA), glycidyl methacrylate (GMA), 2-hydroxyethyl acrylate (HEA), 2-hydroxyethyl methacrylate (HEMA), methyl acrylate (MA), methacrylic acid (MAA), methyl methacrylate (MMA), [3-(methacryloylamino)propyl]-dimethyl (3-sulfopropyl) ammonium hydroxide (MPDSAH) inner salt, N-methyl-N-vinylacetamide (MVA), N-isopropyl acrylamide (NIPAM), N-vinylacetamide (NVA), N-vinyl-caprolactam (NVC), N-vinyl formamide (NVF), N-vinyl-2-pyrrolidone (NVP), 2,4-phenylenediamine (PDA), poly(dimethylsiloxane) (PDMS), poly(ethylene glycol) methacrylate (PEGMA), poly(sulfobetaine methacrylate) (PSBMA), 3-sulfopropyl methacrylate (SPMA), sodium styrene sulfonate (SSS), and trimethylammonium (TMA) to various types of membranes for different separation processes. Membranes made of a variety of polymers such as cellulose triacetate (CTA), PA, PAN, PE, PEEK, PES, polyethylene terephthalate (PET), PP, poly(phenylene oxide) (PPO), PS, PTFE, and PVDF have been endowed with antifouling characteristics through surface modification by RIGC in many studies using different initiation techniques, the details of which are reviewed in the next section.

### 5.1. Membranes Modified with RIGC Using Plasma Treatment

RIGC with plasma technique has been widely used to improve the surface properties of the membranes and enhance their fouling resistance [37]. The majority of the studies applying plasma treatment for antifouling improvements were focused on the treatment of MF and UF membranes made of PES [123,124,125,126,127,128,129], PVDF [130,131,132,133], and PS [134,135,136]. Early studies reported the surface modification of PVDF [137], PE [138], and PES [124] UF membranes, which were modified by RIGC of AAm with plasma for improving hydrophilic surface properties, to overcome the fouling during protein separation. The grafted membranes exhibited better performance, marked by greater flux recoveries after cleaning, revealing reversibility of the protein fouling layer under the influence of the imparted hydrophilicity. PS and PAN UF membranes were modified by RIGC with He-plasmas, with a few monomers such as HEMA, AA, and MAA [134]. In addition, the PES UF membrane used in MBR was also modified by monomers such as AA and HEMA via RIGC with Ar-plasmas [129]. The poly(HEMA) grafted membranes demonstrated higher hydrophilicity and reduced protein fouling compared to the original membranes, and their UF performance improved in terms of both filtrate flux and bovine serum albumin (BSA) retention.

**Figure 7:**
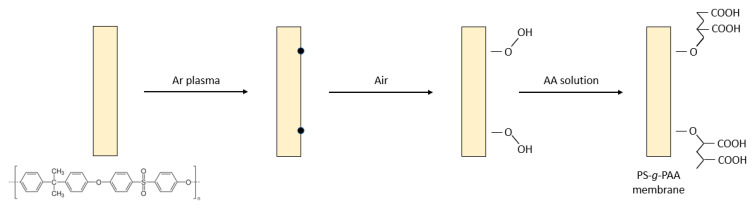
Modification of porous PS membrane by RIGC of AA with Ar plasma treatment.

Early on, Gancarz et al. [135] investigated one of three various methods for imparting hydrophilic character to PS UF membranes by modification with AA, using RIGC initiated by plasma irradiation, as illustrated in Figure 7. The methods included introducing AA from the bulk solution, Ar plasma irradiation and grafting from vapor phase, and plasma polymerization of the monomer vapor in a plasma reactor. The modified membrane surface had a brush-like structure with a strong hydrophilicity and exhibited the most promising protein filtration properties. Two other PS UF membranes, modified by grafting of DMAEMA and AA after treatment with low temperature plasma, were also reported [136]. The former monomer introduced positive charges that reduced the desorption of positively charged lysozyme on the modified membrane, whereas the latter imparted negative charges and reduced the adsorption of negatively charged BSA on the modified membrane.

A long-lasting hydrophilic modification was introduced to PES membranes by Ar-plasma treatment, followed by grafting of AA from a vapor phase, as reported by Wavhal and Fisher [126]. The modified membranes showed highly enhanced pure water flux and reduced protein fouling, in addition to easier recovery of the permeation flux. Meanwhile, Zhao et al. [130] used plasma pre-treatment and graft copolymerization of PVDF powder with AA to make an amphiphilic PVDF-*g*-PAA membrane in a one-pot process. The water flux, BSA rejection, and antifouling ability of the modified PVDF membranes were all improved because of the enhancement of wettability. Moreover, less irreversible fouling of the modified PVDF membrane was observed. The grafting of AA on polymeric membranes enhanced the antifouling properties of the membranes [127]. Positive impacts of AA grafting on the membrane hydrophilicity and antifouling properties have also been reported using other substrates such as PE [139], PTFE [140], PAN [134], and CTA [141].

Commercial PES UF membranes were treated with low temperature He-plasmas followed by RIGC with NVP to enhance the surface hydrophilicity and roughness, as reported by Chen et al. [125]. The surface modified membranes proved to be remarkably less prone to BSA fouling, and the recovery of permeation flux became easier compared to the pristine counterpart. In another study, Zhao et al. [142] prepared PAN NF membranes by modification with low temperature Ar-plasma irradiation and subsequent grafting in NVP aqueous solution to improve the filtration capacity and fouling resistance. The salt rejection from a mixed salt aqueous solution of the poly(NVP) modified PAN NF membranes increased. NVP was also grafted onto PP hollow fiber membranes used in MBR for wastewater treatment. The modification was performed by RIGC with air plasma and the improvement of its limiting flux and antifouling characteristics was reported by Yu et al. [143]. The poly(NVP) modified membranes showed an enhanced filtration behavior in MBR compared to the pristine membrane. Moreover, the relative flux ratio increased by 79% and the flux recovery increased by 53%. The flux was, however, 17.9% lower than that of the pristine membrane. Overall, the modified PP hollow fiber membranes possessed excellent antifouling characteristics. The grafting of NVP on different substrates via RIGC with plasma treatment has enhanced the separation performance and antifouling properties of the membranes for various membrane processes.

Poźniak et al. [144] studied surface modification of PPO UF membranes with sulfonation (monopolar) and combined sulfonation and amine (bipolar) plasma treatment to enhance the hydrophilicity and antifouling properties. The membrane was modified by introducing sulfonic acid groups into membranes using plasma-initiated surface RIGC of SSS in comparison with chemical sulfonation (with H_2_ClSO_3_) of PPO. The bipolar amphoteric membranes with the combined sulfonic acid and allylamine demonstrated not only enhanced filtration capacities but also performed very well in the micellar-enhanced UF of mixtures of the 2,4-D herbicide and hexadecyltrimethylammonium, with 90% removal of 2,4-D herbicide from water. In another study, Li et al. [131] attempted the surface modification of porous PVDF membrane using glycidyl methacrylate-iminodiacetic acid (GMA-IDA) containing carboxylic acid (-COOH) and tertiary amine (-N=) to prepare a bipolar membrane by RIGC using plasma treatment for the conversion of salt and water into acid and base by ED. The ionic groups were subsequently introduced to the grafted membrane by treatment with HCl and NaOH solutions, respectively. After the GMA-IDA monomer was grafted onto the surface of the PVDF membrane, the hydrophilicity of the membrane was dramatically increased. Moreover, the membrane’s ability to separate monovalent and divalent ions was enhanced and fouling within the ED system was reduced. Earlier, the same authors prepared a similar bipolar membrane based on a porous PVDF film that was grafted with AA and DMAEA, using RIGC with plasma treatment [133]. Introduction of anionic and cationic polyelectrolytes to the anion-exchange and cation-exchange layers of the bipolar membranes by RIGC with plasma treatment has improved the membrane hydrophilicity and antifouling properties for the electrochemical processes.

**Figure 8:**
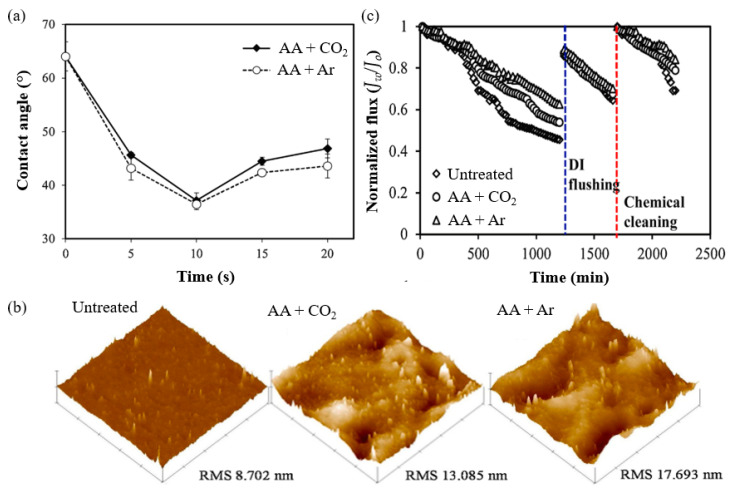
(**a**) Water contact angle, (**b**) 3D AFM images of surface topography, and (**c**) flux decline in BSA filtration. Reprinted from [141] with permission from Elsevier.

A study by Khongnakorn et al. [141] clearly reported the use of LPP treatment to graft AA onto a commercial CTA membrane to improve water flux and impart anti-protein fouling properties in FO for protein recovery. The surface hydrophilicity of CTA membrane grafted with AA in the presence of CO_2_ and the counterpart grafted with AA in the presence of Ar was the highest at the optimum plasma exposure time of 10 s, based on the contact angle results (Figure 8a). The obtained membranes also showed a higher surface roughness, represented by the root-mean square (RMS), as depicted in Figure 8b [145]. The membrane modified with AA in the presence of Ar also demonstrated a significant improvement in the ability to maintain higher water flux over the course of the filtration experiments when compared to the untreated membrane [146]. In a BSA filtration experiment, the untreated membrane suffered the highest flux decline with 55% loss, followed by the membrane grafted with AA in of CO_2_ (50% loss) and that grafted with AA in Ar (36% loss) in the first cycle, as shown in Figure 8c. The flux recovery was achieved after washing with deionized water in the second cycle and chemical treatment in the third cycle. Hence, this study proved that LPP treatment followed by monomer grafting is highly effective for improving membrane anti-protein fouling performance. However, it must be mentioned that LPP treatment is impractical for most industrial applications because it requires a vacuum system, which limits the sample size. Besides, this approach lacks scalability, has high maintenance costs, and system integration is difficult [147].

As an alternative to LPP treatment, APP treatment has been employed for fouling prevention in various applications [45,123,147,148,149,150,151]. APP treatment receives merits over LPP treatment because it can combine UV as an ion bombardment without the need to be in a vacuum space, producing high concentrations of ions and radicals, beside the ability to control the properties of grafted polymer phase [152]. Gu et al. [108] reported that after reacting with oxygen in the air, radicals created on the PES membrane surface by APP treatment were converted into peroxides and could act as initiators for graft copolymerization [108]. As the APP treatment time increased, the number of peroxides increased to the highest amount (4.5 nmol/cm^2^), which is comparable to that obtained using the LPP treatment [153]. The wettability of the membrane was greatly improved by APP treatment, but its antifouling capacity was not significantly improved over time. The possible explanation is that APP treatment failed to maintain the desired surface antifouling properties due to surface restructuring [123]. Extensive APP activation could be used to improve surface hydrophilicity, but this would most likely result in the membrane’s dense skin layer deterioration. Polyethylene glycol (PEG) grafted PES membrane displayed a significantly better protein resistance and antifouling performance than the unmodified and APP treated pristine PES membranes [123]. RIGC using APP treatment has also been applied to modify RO membranes to prevent mineral scaling. Kim et al. [45] utilized a two-step approach whereby a PA TFC membrane was first irradiated with APP, followed by graft copolymerization of MAA monomer. The study showed that the onset time for gypsum scaling on the PA-*g*-poly(MAA) TFC membrane’s surface was delayed, indicating the reduced susceptibility towards mineral scaling compared to a commercial RO membrane. It can be concluded that with optimal APP activation, the RIGC was found to improve the performance of membranes by increasing water flux and minimizing organic fouling or inorganic scaling, depending on the membrane processes and applications.

Chang et al. [154] attempted the application of RIGC using plasma treatment on expanded polytetrafluoroethylene (ePTFE) MF membrane for biofouling prevention. Particularly, a hydrogel-like layer of PEGMA was immobilized on a H_2_ plasma irradiated ePTFE MF membrane. As the grafting degree of the copolymerized PEGMA increases, the hydrophilicity of the surface of the ePTFE MF membranes increases, forming a surface hydrogel-like layer in aqueous solution with regulated coverage. The membrane with low grafting yield exhibited a relative reduction in protein adsorption coupled with a remarkable suppression of platelet adhesion and hemocompatibility. In another study by Dong et al. [155], the PA and polyester membranes modified with PEG via RIGC using plasma treatment showed a substantial reduction in biofouling that was caused by a pathogenic bacteria, *Listeria* *monocytogenes*. The grafting of hydrophilic monomers via RIGC using plasma treatment has been proven to prevent biofouling.

Among the current research trends in curbing biofouling in membrane processes is grafting polymeric membranes with pseudo-zwitterionic functionalities using different techniques [128,132,156,157,158]. Venault et al. [132] reported the use of glow dielectric barrier discharge plasma for inducing the surface grafting of PVDF membranes with two monomers, namely TMA and SBMA. Efficient grafting could not be achieved with SPMA, but it was successful with a combination of SPMA and TMA. The modified PVDF membranes reduced the adsorption of BSA and lysozyme and resisted the attachment of *Escherichia coli*, and thus they were considered very effective in reducing biofouling in static conditions compared to pristine PVDF membrane. High resistance to blood cell and low hemolysis activity showed that pseudo-zwitterionic membranes are compatible with human blood. Apart from that, Jhong et al. [158] prepared ePTFE membranes grafted with zwitterionic PSBMA and PEGMA via plasma-induced RIGC. The ePTFE-*g*-poly(PSBMA) membrane exhibited high wettability and became less adhesive towards protein, human blood, tissue cells, and bacteria. The preparation of membranes with high hemocompatibility and biocompatibility and low biofouling by RIGC with zwitterionic monomers using plasma treatment could be very beneficial for hemodialysis application.

An example of modifying a polymeric membrane surface with zwitterionic monomer by RIGC using air plasma (corona) treatment for flux enhancement and fouling reduction was reported recently by Salimi et al. [128]. SPMA was grafted onto the surface of a PES membrane, leading to desired grafting yields by controlling grafting conditions. The grafted membrane displayed a maximum increase of about 1100% in permeate flux for oil/water emulsion filtration compared to unmodified membrane. Moreover, the pure water flux increased by up to 1000%, together with a maximum flux recovery ratio enhancement of 180%, which is a significant improvement. Despite its scarcity, the use of corona treatment followed by grafting of monomer has been reported to yield less damage to membrane bulk and pore structure compared with other plasma treatments and, most importantly, enhance the antifouling properties of the membranes [127,128,129,139].

RIGC using plasma treatment has plenty to offer, whereby its impact on the improved antifouling properties of polymeric membranes is significant for various membrane processes. When compared to non-modified membranes, the modified membranes attained lower fluxes but with higher flux recoveries. Furthermore, this method needs a very short time to modify the membrane surface. The type of plasma process, whether it is LPP or APP, and the selection of plasma gas, which controls the grafting yield, are among the parameters that made this modification method effective. The previous studies, which addressed various modifications of polymeric membranes via RIGC using plasma treatment for fouling prevention, are summarized in Table 3.

### 5.2. Membranes Modified with RIGC Using UV Treatment

RIGC using UV treatment is suitable for modification of intrinsically photoactive polymeric membranes. This approach requires either a photo-sensitive base polymer, such as PES and PS, or the introduction of photo-sensitive groups onto the membrane surfaces prior to graft copolymerization, and involves the direct generation of free radicals from polymer substrate under UV irradiation [83].

The earlier work by Pieracci et al. [159] reported modification of PES UF membranes by RIGC with NVP, NVF, and NVC using UV irradiation to obtain hydrophilic membranes with low fouling surfaces. The membrane modified with poly(NVP) showed a 25% increase in the hydrophilicity, a 49% decrease in BSA fouling, and a 25% increase in BSA retention, compared to untreated PES membrane. Moreover, this membrane attained the best combination of low fouling and high flux among all tested membranes. UV-assisted RIGC of NVP onto 50 kDa PES UF membranes was also investigated, using two different techniques involving dip modification and immersion modification, by the authors [160]. The grafted PES membranes showed highly wettable surfaces with superior fouling resistance compared to the pristine membrane. The membranes grafted by all the above modification techniques were prone to simultaneous loss of BSA rejection and permeability, and the level of such loss depended on the grafting level. This was due to the pore obstruction caused by invaded grafted polymer chains, and such effect was predominant at high poly(NVP) concentrations, suggesting that, as radiation cleaved PES bonds and enlarged the pores, a high density of long chains was created on the surface. The wettability and fouling data indicated that the irreversible adsorptive fouling can be eliminated by modifying the base membrane with poly(NVP) at low grafting yield, but with a negative impact on the permeability. On the other hand, higher grafting yield causes a further loss in the permeability. Pieracci et al. [161] extended their work to further improve the permeability of the poly(NVP) grafted PES UF membrane, using 2-mercaptoethanol as a chain transfer agent followed by ethanol cleaning. The increased concentration of chain transfer agent reduced the graft chain density and length. Meanwhile, the non-grafted homopolymer that initially blocked the membrane pores was removed by ethanol due to membrane swelling. Consequently, the permeability of the PES UF membrane improved, but at the expense of losing protein rejection. It was suggested that the incorporated poly(NVP) grafts promote the swelling and pore enlargement of the membrane, hence causing severe loss of protein rejection. On the contrary, the incorporation of poly(NVP) grafts on PES and sulfonated PES NF membranes via RIGC using UV treatment by Kilduff et al. [162] significantly reduced fouling caused by natural organic compounds, while maintaining pure water permeability and solute rejection at the levels comparable to those found in neat PES NF membranes.

**Figure 9:**
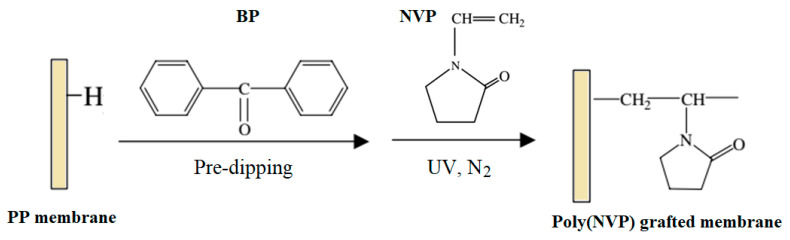
Modification of porous PP membrane by RIGC of NVP with UV irradiation. Reprinted from [163] with permission from Elsevier.

A porous PP MF membrane prepared by UV-assisted RIGC of NVP showed lower protein adsorption and platelet adhesion and more hemocompatibility with the increased grafting yield [163]. PP MF membranes were also modified by RIGC with NVP using UV radiation under various reaction conditions, as illustrated in Figure 9. The increase in the monomer concentration (10–70 vol%) and treatment time showed minor effects on grafting yield of poly(NVP) on PP MF membrane. It was suggested that UV irradiation was limited for RIGC initiation of NVP on the membrane surface [163]. In another study, PP membrane with novel antibacterial properties comprising surface immobilized poly(NVP)-iodine complex was also reported by Xing et al. [164]. The NVP was grafted onto PP membranes using RIGC with UV irradiation followed by complexation of iodine on poly(NVP) grafted membrane. The content of iodine could be adjusted by controlling grafting, which could be manipulated by varying the irradiation time or the monomer concentration. The obtained membrane was proven to have efficient antibacterial activity against *Escherichia coli*, *Staphylococcus aureus*, and *Candida albicans*.

PP membranes were also modified with other hydrophilic monomers using the same RIGC method for fouling prevention [165,166,167,168]. For example, Gu et al. [165] modified PP microporous membranes with sugar-containing monomers, such as GAMA. The antifouling properties of the membrane improved dramatically during the MBR as the grafting chain length was increased. The highly hydrated poly(GAMA) layer grafted on the membrane surface prevented the foulant adhesion and, as a result, the foulant could be easily removed by water washing, imparting this membrane with reversible fouling characteristics. In another study, Hu et al. [166] used HEMA to improve the surface hydrophilicity of a PP microporous membrane with the addition of FeCl_3_ and BP. The former acted as an inhibitor for homopolymerization, whereas the later was a photo-initiator. This increased grafting yield and imparted a remarkable enhancement of the hydrophilicity of the membrane, leading to better antifouling and hemocompatibility than the neat PP membrane. Apart from that, grafting of AAm on PP membranes via RIGC with UV treatment also improved the hydrophilicity and flux recovery of the PP-*g*-poly(AAm) membranes [167,168].

Other polyolefin membranes, such as PE membranes, were also modified, whereby hydrophilic monomers such as 2-methacryloyloxyethyl phosphorylcholine (MPC), NVP, AAm, and methacryloyl poly(ethylene glycol) (MPEG) were graft-copolymerized onto PE membrane using UV irradiation [164]. Of all monomers, the incorporation of poly(MPC) was found to significantly improve protein adsorption together with the platelet adhesion resistance, and so the NVP grafted membranes were compared to pristine PE membrane. Surprisingly, membranes incorporated with poly(AAm) and poly(MPEG) did not show any effect on protein adsorption. PE membranes that are hydrophilic, electrically neutral, and have smooth surface are less likely to be fouled [169].

Selecting the right monomers for surface modification of polymer substrates via RIGC using UV treatment is important to achieve desired membrane properties. In several attempts to prepare membranes with low biofouling properties, hydrophilic monomers such as quaternized 2-(dimethylamino) ethyl methacrylate (qDMAEMA), AMPS, and HEMA possessing respective basic, acidic, and neutral natures were used to modify PES and PVDF membranes [170,171,172]. The membranes modified with qDMAEMA were found to have distinct antibacterial surface properties in a way that they stopped microorganisms’ growth, reducing in the formation of fouling biofilms. The membranes showed a strong antimicrobial effect against *Escherichia coli*, and this biocidal property enhanced the resistance to biofouling in water treatment applications. In another study, MPC was grafted on a PEEK membrane surface via RIGC using UV treatment to prepare membranes with high wettability and low protein adsorption for medical applications [173]. Taniguchi et al. [174] tested six different hydrophilic monomers: NVP, HEMA, AA, AAG, SPMA, and AMPS for modification of PES UF membranes with RIGC using UV treatment for natural organic matter removal. The AA grafted membrane displayed the most stable filtration over a long period and recorded the lowest fouling with zero irreversible fouling. Evaluation of membranes grafted with these monomers for BSA rejection suggested that AA was the most sensitive to UV oxidation and copolymerization. Thus, AA has been mostly selected to modify polymeric membranes by RIGC, using UV treatment for various membrane processes [175,176,177]. A small increase in the wettability of AA grafted membrane was enough to prevent irreversible fouling, whereas the high swelling imposed by NVP and HEMA caused reduced BSA rejection, despite the improvement in reducing fouling compared to the commercial PES UF membrane [178]. Grafting of AA for fouling prevention showed superiority compared to grafting of amino monomers (AAm, EDA, PDA) or other acrylic monomers (HEMA, PEGMA) when using different polymer substrates such as PAN [179], PP [168], and PVDF [180] membranes. However, there was also a study that reported the higher flux recovery ratio of PEGMA grafted PES nanoporous membranes compared to grafting with AA, PDA, and EDA monomers [181], whereas another study on polyimide (PI) UF membranes showed otherwise [182]. In a comparison study between PEG and three monomer pairs (PEG–NVF, PEG–NVA, and PEG–MVA) for modification of PES via RIGC using UV treatment, PEG–NVA was the best monomer pair grafted on the PES membrane to resist fouling by BSA, proving that binary systems can improve the protein resistance of a membrane [183].

Helin et al. [184] grafted a PS UF membrane with MA by RIGC using UV treatment. The increase in the grafting yield improved the hydrophilicity of grafted membranes, and the results of pure water and BSA solution permeation proved that adding MA to the PS structure improves the membrane’s antifouling property. Similar outcomes were reported by Yu et al. [185], who incorporated a zwitterionic molecule, MPDSAH on a PS UF membrane. The hydrophilicity of the membrane was improved while showing superior separation and consistent pure water flux. The MPDSAH grafted membranes outperformed the PS UF membrane in terms of antifouling properties in the pH range of 4.5–10.0. In contrast, a PES UF membrane modified by RIGC with PEGMA using UV treatment outperformed the membranes modified by new generation material, zwitterionic N,N-dimethyl-N-(2-methacryloyloxyethyl-N-(3-sulfopropyl)ammonium betaine (MMESPAB), in terms of fouling resistance [186].

In a recent study to develop an anti-coagulation PET membrane surface for blood filtration [187], a photoactive pseudo-zwitterionic copolymer (PZC) made up of 2-carboxyethyl acrylate, trimethyl-2-methacroyloxyethylammonium chloride, and 2-methacryloyloxyethyl-4-azidobenzamide was first synthesized by free radical polymerization under Ar atmosphere. A PET substrate was then immersed in the PZC solution, followed by irradiation with UV to produce PZC grafted PET membrane. The presence of copolymerized PZC in the fibrous membrane structure with a balanced composition of cationic and anionic groups resulted in excellent anti-coagulation surfaces. The PZC grafted PET membrane, prepared by RIGC using UV treatment, was able to prevent platelet adhesion and activation. Weinman et al. [188] first developed a novel zwitterionic polymer, namely poly(2-((2-hydroxy-3-(methacryloyloxy)propyl)dimethylammonio)acetate)(poly(CBOH)), to modify PES UF membrane via UV-induced graft copolymerization for reduced biofouling. The modified membrane had a unique property whereby its surface chemistry may switch between the anti-fouling, zwitterion mode and an anti-microbial, quaternary amine mode by changing the pH. The preparation of membranes with low biofouling can be achieved by RIGC with zwitterionic polymers using UV treatment.

In a study by Mondal and Wickramasinghe [189], a commercial PA TFC membrane was grafted with NIPAM using RIGC with UV irradiation to prepare an antifouling NF membrane with a relatively high salt rejection. BP was initially adsorbed onto the membrane surface by immersion in its ethanol solution followed by grafting with NIPAM. The modified membrane attained a significant hydrophilicity increase because of the formation of poly(NIPAM) hydrogel layers that vary in density depending on reaction parameters. Despite experiencing reduced water flux, the formation of a thick hydrogel mat on the membrane grafted surface resulted in a huge increase in salt rejection and fouling resistance compared with the commercial NF membrane. Moreover, the grafted membrane surface could release the accumulated foulants on the membrane surface when flushed with warm water, thanks to the temperature responsive characteristic of poly(NIPAM) [190,191]. According to this study, changing the membrane’s surface chemistry by RIGC of poly(NIPAM) using UV treatment has significantly reduced the negative effects of colloidal fouling in NF.

**Figure 10:**
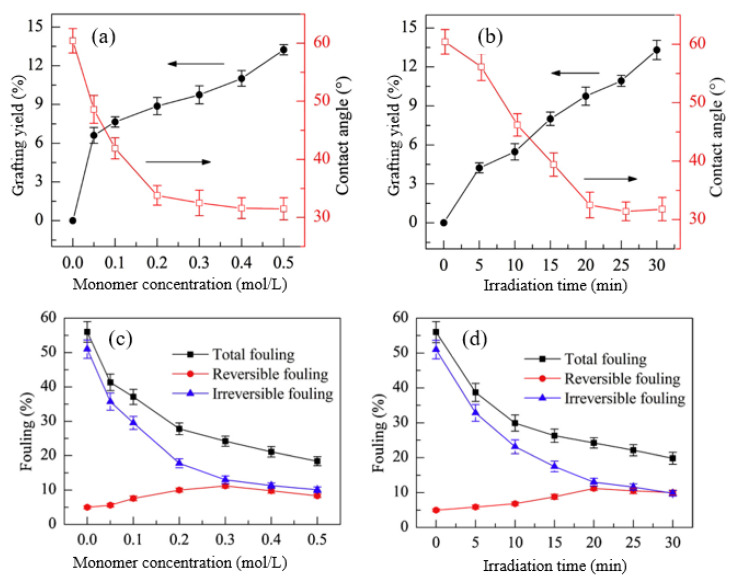
The effects of HEA concentration (irradiation time = 20 min) on: (**a**) grafting yield and contact angle and (**c**) fouling and the effects of irradiation time (HEA concentration = 0.3 mol/L) on (**b**) grafting yield and contact angle and (**d**) fouling. Reprinted from [99] with permission from Elsevier.

Huang et al. [99] fabricated a PEEK UF membrane, with a surface modified by HEA as a hydrophilic monomer through UV induced graft copolymerization under various reaction conditions, to control not only the grafting yield and surface hydrophilicity but also surface morphology and fouling resistance. Particularly, the monomer concentration and irradiation time were used to control the grafting yield, contact angle (hydrophilicity), and fouling, as shown in Figure 10.

Figure 10a shows the impact of monomer concentration and UV irradiation time on grafting yield and surface hydrophilicity. The increase in both HEA concentration and reaction time caused an increase in the grafting yield, which consequently increased the hydrophilicity, as indicated by the reduction in the contact angle. This suggests that the change in the properties of modified membranes is a function of grafting yield which is heavily dependent upon reaction conditions. A similar trend was also reported in a study by Zhang et al. [192], in which NVP was grafted on a PVDF/PES MF membrane using UV irradiation under various monomer concentrations and reaction times. The improved hydrophilic property due to increased monomer concentration and irradiation time has gradually reduced the total fouling and irreversible fouling caused by hydrophobic molecules, as indicated in Figure 10b, which shows the small increase in reversible fouling after grafting of HEA [193]. This proves that the hydrophilic HEA chains help to weaken the membrane-BSA interaction during cleaning, thus allowing the flux to increase. These findings show that increasing grafting density and graft chain length can improve antifouling performance, which is consistent with the previous research. [194,195]. Prolonging the irradiation time produces more active sites and increases graft initiations, and thus increases the graft chain number [185]. However, furthering irradiation time to a longer extent can lead to excessively long graft chains, causing membrane pore blocking and permeability reduction. In another study, Susanto et al. [196] prepared low fouling UF membranes made up of PES that was modified by PEGMA through RIGC with UV irradiation. The results revealed that the most important parameter for adjusting the degree of functionalization was UV irradiation time, followed by monomer concentration. Although the membranes showed higher fouling resistance and rejection, pore constriction or even blocking by grafted poly(PEGMA) has resulted in a decrease in permeability. Therefore, it can be concluded that the increase in the grafting density endows a rise in the hydrophilicity and improves the membrane permeability, unlike the growth in the grafted chain length, which leads to pore blockage and reduction in the membrane permeability [197].

Xueli et al. [198] prepared BHMBA grafted PS UF membrane using RIGC UV irradiation in the presence of BP. Despite the addition of BP, the grafting yield of the grafted membrane only increased by 24% without significant changes in the surface roughness, antifouling property, and antibacterial efficiency compared to those of the membrane grafted in the absence of BP. One plausible reason is that the grafting took place only on the upper membrane surface, not on the membrane pores [199]. The main disadvantage of this approach is the dependence on BP concentration; the low concentration of BP at the membrane surface leads to a low grafting efficiency, whereas a high bulk BP concentration in the monomer solution can lead to homopolymerization. Thus, optimization of the concentration of photo-initiator concentration together with other reaction parameters is essential for effective membrane modification by RIGC with UV irradiation. The various modifications reported for polymeric membranes via RIGC using UV irradiation for fouling prevention are summarized in Table 4.

### 5.3. Membranes Modified with RIGC Using γ-rays

Compared to UV and plasma treatments, γ-rays have a high penetration depth and strong energy that can produce radicals at the inner parts of the polymeric materials, regardless of their thickness. Due to this advantage, γ-rays are suitable for preparation of pore-filled membranes, integrating the mechanical strength of the substrate with the high conductivity of polymer electrolyte for ion exchange applications, such as fuel cell [200], ED [201], and RED [202]. Nevertheless, several studies considered the utilization of this graft copolymerization technique for pressure driven membrane processes, which makes this technique, as applied to fouling prevention, appealing [203,204,205,206,207,208,209,210,211,212,213]. The early study by Shim et al. [209] reported the modification of the surface of porous PP membranes by RIGC of HEMA using γ-rays. The grafting yield reached up to 76% by optimizing the absorbed dose and reaction time. The modified membranes acquired an increased hydrophilicity and showed a decrease in the water flux due to the narrowed and plugged pores with grafted poly(HEMA). The rise in hydrophilicity resulted in a maximum two-fold increase in BSA solution flux. After deionized water and chemical cleaning, the flux recovery of the modified membranes was superior to that of the unmodified membrane. It was deduced that the grafted poly(HEMA) chains prevented the hydrophobic interactions between BSA molecules and the membrane surface and reduced BSA adsorption. As a result, the modified membrane acquired an improved antifouling and washing properties.

In another study, Sehgal and Rattan [205] studied the RIGC of MMA onto irradiated isotactic PP membrane via peroxidation method, starting with γ-rays’ irradiation from a Co60 source to fabricate a PP-*g*-poly(MMA) membrane. The reaction conditions were manipulated to promote graft copolymerization over homopolymerization, which is very possible because of monomer’s high reactivity. The latter was suppressed by the addition of a small amount of FeCl_3_ as an inhibitor. The obtained membrane acquired good hydrophilicity and remarkable swelling behavior in different solvents. A similar isotactic PP membrane grafted with NVP using the same RIGC method was also reported by the same authors, who obtained membranes with improved hydrophilicity and pH sensitivity [214]. NVP was also grafted on a PP MF membrane using RIGC with γ-rays and UV radiation. The pre-irradiation with γ-rays was proven to be more efficient with respect to grafting yield. The observed reduction in the protein adsorption and platelet adhesion provided evidence for the enhancement of and hydrophilicity and hemocompatibility by incorporation of poly(NVP) chains to the PP membrane. The water flux increased at low grafting yield, reaching a maximum of 7.3 times higher than the pristine membrane. A higher grafting yield undermined the water flux, which was a result of competition between the hydrophilicity and the variation in pore size of the grafted membrane [163].

Deng et al. [213] investigated the application of RIGC of MAA on PES powder using γ-rays to prepare PES MF membranes by polymer solution casting. The membrane attained an increasing hydrophilicity as a function of grafting yield. Consequently, the swelling was increased and pore size was enlarged, leading to an improved filtration flux. The pH value of the aqueous solution had no effect on the properties of the MF membranes made from neat PES. On the contrary, the MF membranes fabricated from the poly(MAA) grafted PES powder were pH dependent. The increased degree of swelling decreased both pore size and flux, which were attributed to the increase in the ionization of the grafted poly(MAA) side chains with increasing alkalinity of the solution. A similar PES membrane, but in a hollow fiber form with hemocompatibility properties, was also investigated by Wang et al. [206]. The membrane was prepared by RIGC of SSS, AA, and NVP on PES substrate using γ-rays. Interestingly, there was no platelet adhesion observed on the PES-*g*-poly(AA/NVP/SSS) membrane. Furthermore, the amounts of BSA and bovine fibrinogen (BFG) deposited on the membranes showed a remarkable decrease for PES-*g*-poly(AA/NVP/SSS) membrane compared to PES-*g*-PVP membrane. In addition, the modified membranes did not cause hemolysis or activate complement, and the blood clotting time was slightly delayed. These results confirm the effective endowment of the anti-platelet adhesion property and hemocompatibility to PES membranes, which became more suitable for hemodialysis application.

**Figure 11:**
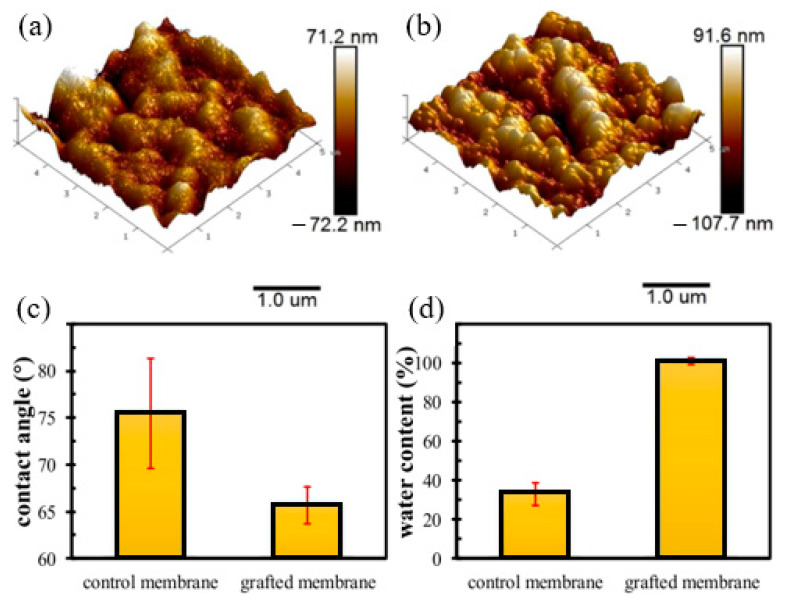
AFM images, contact angle, and water content of the control and grafted membranes: (**a**) AFM image of the control membrane; (**b**) AFM image of the grafted membrane; (**c**) contact angle; (**d**) water content. Reprinted from [215], published by Nature.

One of the advantages of RIGC using γ-rays is that it allows bulk grafting or modification of the membrane core, and therefore dramatic changes in the physico-chemical properties, such as hydrophilicity and wettability of the whole membrane, can be introduced. To discuss the impacts of such changes on fouling prevention, a study was carried out by Shen et al. [215] to modify PVDF membrane by RIGC with HEA using γ-rays, and the properties of the prepared PVDF-*g*-poly(HEA) membrane were evaluated. The copolymerization of HEA on the PVDF membrane produced a membrane with a rougher surface and higher hydrophilicity compared to the control membrane, as indicated by the AFM images and water contact angle results shown in Figure 11a–c. Furthermore, the water content ratio of the grafted membrane was three times higher than that of the control membrane (Figure 11d), indicating that the grafted membrane has significantly improved in its wettability. The substantial increase in membrane wettability has proven that RIGC produces a homogeneous membrane structure with grafted hydrophilic polymer chains across the membrane.

The grafted chain matrix on the membrane had a higher tendency to experience configurational change or swelling due to the chemical structure of the grafted membrane that absorbs more water molecules [195]. Swelling of the chain matrix which can be affected by either pH or ionic strength of the solution would reduce the pore size, leading to regulation of the water flux [215,216]. It was found that the flux of the grafted membrane has a strong dependence on pH in the acid-stage filtration. At a neutral pH, the grafted HEA chains tend to swell because the chemical potential of the entire matrix is the lowest in this state. Considering the solution’s ionic strength, high ionic strength results in high electric double layer compression, which helps to resist swelling of the grafted chain matrix.

**Figure 12:**
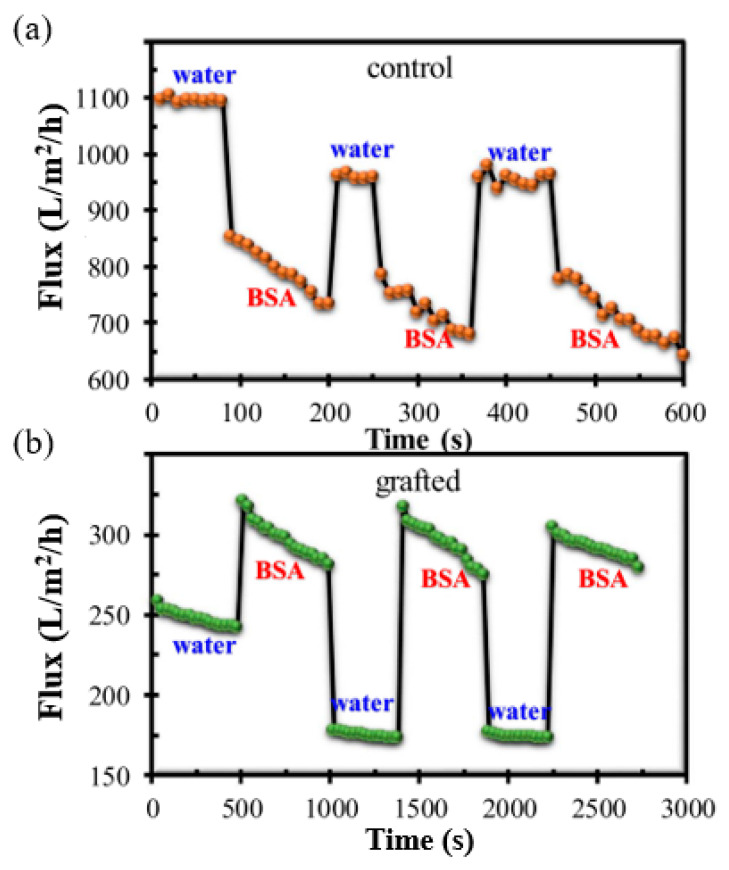
Antifouling performance of membrane by alternate filtration of pure water and BSA solution: (**a**) flux of control membrane and (**b**) flux of grafted membrane. Reprinted from [215], published by Nature.

The impact of this adjustable pore size on the antifouling property of the grafted membrane is shown in Figure 12. The grafted membrane exhibited a lower water flux compared to the control membrane, caused by the swelling of the grafted chain matrix in pure water without ionic strength, resulting in a significant reduction in the pore size. When the pure water was replaced by BSA solution with the same pH value of 7.0, the flux of the control membrane decreased significantly. The flux profile versus filtration time shows that the control membrane experienced severe fouling after only a short period of filtration. In contrast, the PVDF-*g*-poly(HEA) membrane demonstrated a greater value of BSA solution flux than that of pure water in three complete filtration cycles. The BSA solution’s relatively high ionic strength prevented the swelling of the grafted chain matrix and enlarged the membrane’s surface pore size, and thus, the BSA flux was found to be greater than the water flux of the grafted membrane. The flux’s decreasing rate demonstrated unequivocally that the grafted membrane had greater antifouling ability.

To further improve the antifouling ability of PVDF membranes, a new strategy for grafting HEA through γ-rays radiation was proposed by Shen et al. [212] by conducting thermodynamic analyses. The grafted membrane possessed an improved antifouling ability for filtration of sodium alginate solution, which was experimentally evidenced by the increased flux recovery ratio and the decrease in the irreversible fouling ratio. The results suggested that the improved antifouling performance was primarily due to improved hydrophilicity and reduced strength of thermodynamic interactions between the grafted membrane and foulants.

**Figure 13:**
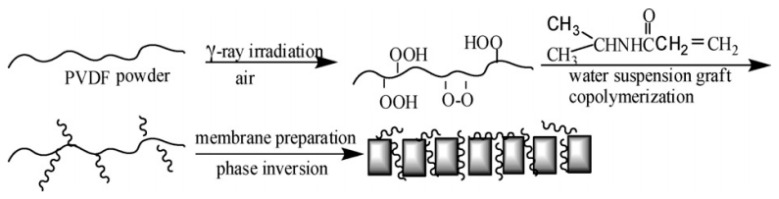
Modification of PVDF powder by RIGC of NIPAM with UV irradiation, followed by membrane preparation by phase inversion. Reprinted from [204] with permission from Elsevier.

Other hydrophilic monomers such NVP [203,208], polyvinyl alcohol (PVA) [211], and NIPAM [204] were also grafted on PVDF membranes for fouling prevention. In the preparation of PVDF-*g*-poly(PVA), the PVA was directly anchored onto PVDF membrane surfaces via RIGC with γ-rays irradiation [211]. The modified PVDF membrane attained a high hydrophilicity and underwater superoleophobicity, which improved the anti-oil fouling and cleaning properties of the membranes. Qin et al. [203] prepared PVDF MF membrane by modification with NVP using RIGC with γ-rays. The grafting yield increased as the NVP concentration, absorbed dose, or dose rate increased. This caused an increase in both surface hydrophilicity and surface roughness coupled with a decrease in the pore size. Furthermore, the membrane’s water uptake and water flux were also improved. It was proven that the modified membranes showed better antifouling properties. Similar trends were observed in a study by Yu et al. [204], whereby an amphiphilic copolymer, PVDF-*g*-poly(NIPAM), was synthesized via RIGC using γ-rays, followed by phase inversion to produce membranes (Figure 13). The membrane water flux increased drastically when the concentration of the grafted copolymer chains increased [204]. This trend was caused by the pore-forming ability of the amphiphilic additive that enhanced the membrane hydrophilicity.

The recent work by Li et al. [210] highlighted the modification of PA TFC RO membranes by PVA via coupling of interfacial polymerization with RIGC using γ-rays. The incorporation of poly(PVA) grafts improved the salt rejection of the RO membrane by increasing the degree of crosslinking of the separation layer, hence strengthening the steric hindrance. Consequently, the prepared membrane acquired a salt rejection efficiency of 99.4%. This was accompanied by an improvement in surface hydrophilicity. In addition, poly(PVA) grafts gave the membrane excellent antifouling properties, as demonstrated by the BSA fouling test and the reduced irreversible flux decline ratio.

Thus, it can be concluded RIGC using γ-rays is rather unique compared to the use of plasma and UV treatments, in the sense that it allows bulk grafting depending on irradiation dose rate and absorbed dose. Therefore, the number of studies reporting the use of γ-rays for the modification of polymeric membranes via RIGC for fouling prevention has increased in the past decade. A summary of previous studies is presented in Table 5.

### 5.4. Membranes Modified with RIGC Using EB

RIGC using EB is a simple and very fast technique that allows surface as well as bulk grafting depending on the acceleration energy of electrons and, thus, it is a distinctive facility for the modification of membranes and films [150,217] During the early application of RIGC with EB for fouling prevention of polymeric membranes, a binary mixture of SSS and AA was grafted to improve the hydrophilicity of PVDF and PTFE membranes [218,219]. Particularly, Liu et al. [218] modified PVDF membranes with high-energy EB using pre-irradiation (under vacuum), followed by a single step grafting with mixture of AA and SSS to introduce hydrophilic character to the membrane. This was evident from the reduced water contact angle of the modified PVDF compared to the pristine membrane.

**Figure 14:**
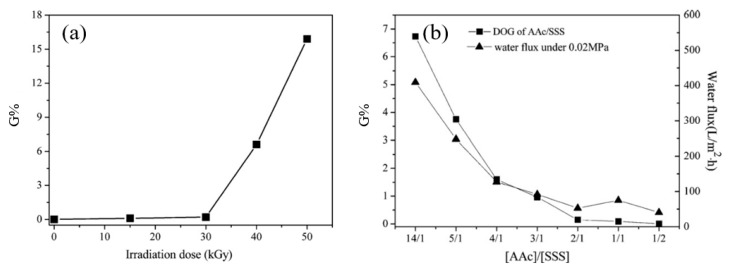
Effect of reaction conditions on the grafting yield: (**a**) effect of irradiation dose and (**b**) effect of AA content in binary monomer mixture [AA/SSS]. Reprinted from [219] with permission from Elsevier.

In another study, Xi et al. [219] introduced the hydrophilic sulfonate groups by a single step grafting method with binary monomer solution of AA and SSS on PTFE porous membranes, pre-irradiated by EB under vacuum. The grafting yield was found to be dependent upon the irradiation dose and the AA content in the binary monomer, as shown in Figure 14. The presence of AA made it possible to graft SSS, which is difficult to graft due to its strongly hydrophilic nature [206,218,219]. Figure 14b shows that, as the AA content in the binary monomer increases, the grafting yield increases. The grafted membrane acquired strongly hydrophilic character unlike the strongly hydrophobic neat PTFE counterpart.

**Figure 15:**
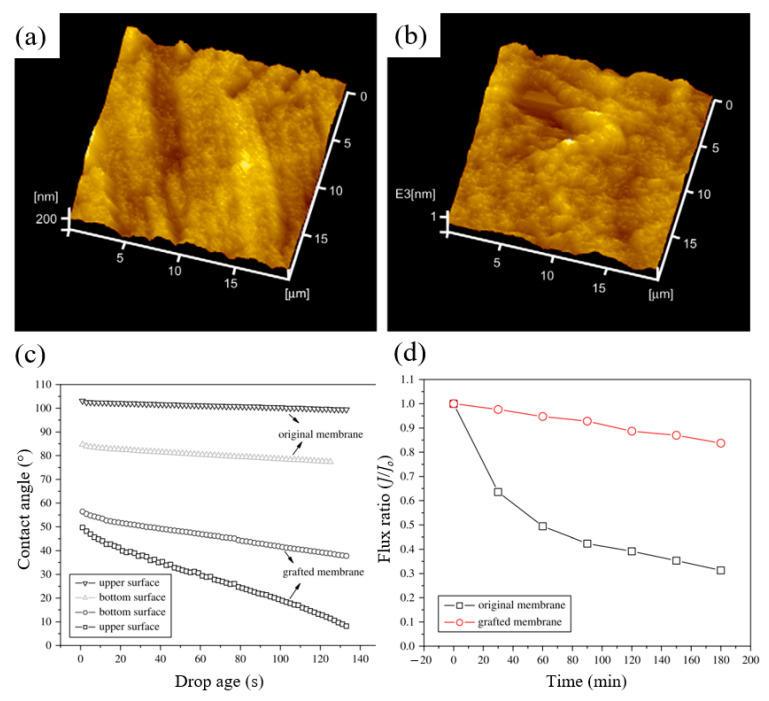
3D AFM images of surface topography of: (**a**) original membrane; (**b**) grafted membrane; (**c**) water contact angle and (**d**) flux ratio of 1.0 g/L BSA solution to the initial pure water flux. Reprinted from [220] with permission from Elsevier.

In another study by Liu et al. [220], grafting of PEGMA onto PVDF surface by RIGC using EB was conducted, whereby the factors affecting the grafting yield were investigated. The rise in the concentration of PEGMA monomer led to a rapid increase in the grafting yield, which was affected by solution pH. The high pH made PEGMA hydrophilic, increased viscosity of the solution, and became rather incompatible with hydrophobic membrane surface, which inhibited monomer diffusion onto the PVDF membrane surface and slowed the grafting process [221]. In contrast, the PEGMA monomer had a hydrophobic structure at lower pH, which made it more compatible with the hydrophobic PVDF membrane, leading to rapid monomer diffusion and an increase in the grafting yield. The maximum grafting yield was 21%, obtained at pH 1.0 of the monomer solution. RMS decreased from 5.64 to 1.84 nm by grafting due to the presence of the grafted poly(PEGMA) brushes, according to 3D AFM images of surface topography shown in Figure 15a. Grafting also affected the hydrophilicity of the membranes, as shown in Figure 15b, whereby the contact angles of the grafted membrane decreased significantly in the presence of poly(PEGMA) brushes. The hydrophilic nature of the poly(PEGMA) side chains significantly improved the hydrophilicity of PVDF membranes.

The effect of grafting on the degree of fouling is shown in Figure 15c. In 3 h, the flux of the original membrane dropped to about 30% of the initial pure water flux, while the flux of the grafted membrane had maintained more than 85% of the initial pure water flux. The grafted hydrophilic poly(PEGMA) chains weakened the hydrophobic interactions between BSA molecules and the PVDF membrane, thus affecting BSA surface adsorption.

The application of RIGC using EB was also performed on sulphone-based polymer substrates for fouling prevention. Schulze et al. [222] modified the surface of PES membranes by immersing in the solutions of the low-molecular weight monomers with different hydrophilic functionalities: carboxylic, sulfonic and phosphoric acids, amines, alcohols, and zwitterionic compounds, followed by EB treatment. Results showed that the modified membranes experienced a substantial reduction in the albumin and myoglobin adsorption. In another study, a simultaneous RIGC with EB was employed to prepare PS NF membranes with high negative charge density for chromium ion, Cr(VI), and removal [223]. AMPS monomer was grafted on both outer and inner surfaces of a PS substrate. The Na_2_SO_4_ rejection was improved at the expense of losing permeate flux as the negative charge density increased, due to the smaller mean pore size of membrane. Cr(VI) was successfully removed from an alkaline aqueous solution by the modified NF membrane. With a permeate flux of 23.8 Lm^−2^h^−1^ at 4 bar and pH 9.0, the membrane grafted under 80 kGy with 10% AMPS solution showed 95.1% rejection of Cr(VI). Interestingly, the presence of Na_2_SO_4_ and NaCl in the experimental range posed no effect on Cr(VI) removal performance, thus reducing the propensity for inorganic scaling problems.

By applying RIGC by EB, grafting of hydrophilic polymers, which include PEG, pluronic (PLU), PVA, PVP, polyallylamine hydrochloride (PAH), and polystyrene sulfonate (PSS), was performed on PVDF membranes for biomedical applications [224]. The modified PVDF membranes showed better wettability compared to the pristine counterpart. Based on contact angle results, grafting of PEG, PLU, and PSS yielded a high hydrophilicity. In addition, the irradiated membranes did not cause hemolysis or coagulation when incubated with whole blood. Overall, PVP modification produced the best results in terms of the membrane stability due to the decrease in contact angle and blood coagulation. Schulze et al. [217] also covalently attached a digestive enzyme, trypsin, to the surface of a PVDF MF membrane by a one-step RIGC using EB. The biocatalytic membranes showed significantly improved antifouling properties compared to the neat PVDF MF membrane. Moreover, trypsin could be controlled by changing pH to actively degrade a fouling layer of proteins. The modified membrane restored 90% of the initial water permeation flux, whereas the neat membrane recovered only 27%. It can be concluded that the RIGC using EB did not produce any toxic breakdown products or long-term reactivity, hence ensuring its safe utilization for biomedical applications.

In a study by Nguyen et al. [225], a commercial PVDF/PVP membrane was irradiated at 10 and 100 kGy with EB in the presence of zwitterion L-cysteine, phosphocholine, and DMAEMA to enhance the fouling resistance of the membrane. A smoother surface and smaller pore sizes were achieved in the modified membranes with the zwitterion compound, accompanied by the improved antifouling capacity exhibited by the lower flux decline and prominent flux recovery. Compared to the neat and irradiated PVDF/PVP membranes with a 10 kGy absorbed dose, the irradiated PVDF/PVP membranes with a 100 kGy absorbed dose showed lower initial fluxes. The 10 kGy irradiated PVDF/PVP membrane displayed the best fouling resistance in the presence of L-cysteine.

Shawky et al. [226] reported the modification of poly(vinylidene fluoride)-*co*-hexafluoropropylene (PVDF-*co*-HFP) NF membrane by incorporation of povidone-iodine complex. This was carried out with RIGC of NVP using EB and subsequent I_2_ immobilization. The obtained membrane showed an excellent antimicrobial activity in the form of a complete inhibition (killing, not filtering) of the bacterial growth against *Escherichia coli* and *Staphylococcus aureus*, compared to the pristine and (PVDF-*co*-HFP)-*g*-PVP membranes. The I_2_ immobilized membrane also demonstrated remarkable improvement in pure water permeation flux and bacterial filtration through the membranes, indicating the reduced biofouling.

Recently, Lim and Shin [25] modified PVDF membranes via RIGC using EB with GMA/EDMA binary monomer followed by a sulfonation process. The grafted PVDF membranes with EDMA produced a denser structure, which reduced the amount of oxirane groups converted to sulfonic groups. The modified membrane demonstrated the highest ion exchange capacity (1.61 meq/g) when grafted with 0.5 *w*/*w*% GMA in the absence of EDMA. This membrane showed the highest level of hydrophilicity among other modified membranes. In terms of filtration, the negatively charged membranes had higher water permeability because of their electrostatic repulsion and sieving effect. The electrostatic repulsion between the membrane and foulants also helped to reduce membrane fouling. It was found that the use of binary monomer in this study is unnecessary, as the presence of EDMA only limits the modification of the PVDF-*g*-poly(GMA) membrane.

Despite its merits, the RIGC technique using EB has not been fully utilized for fouling prevention until recently. This is likely caused by captivity of most of the EB accelerators in radiation research institutes or relevant industries, where they are dedicated for routine production. Because of the speed and the absence of any hazardous material during surface modification and the high stability of the grafted membrane, this method is thought to be suitable for the modification of polymeric membranes, not only for typical pressure driven membrane processes, but also for medical applications such as hemodialysis, with improved membrane hemocompatibility and biocompatibility. The modification of polymeric membranes via RIGC using EB for fouling prevention is summarized in Table 6.

### 5.5. Membranes Modified with RIGC Containing Metal Nanoparticles

The use of nanoparticles to change the surface properties of membranes with respect to hydrophilicity and charge for imparting antifouling functions to the obtained nanocomposite membranes has been widely investigated [227]. Various metals such as Ag and Cu [228] and metal oxides such as Fe_2_O_3_, CuO, TiO_2_, and ZnO [229] have been used for the development of membranes with antimicrobial characteristics. Modification of membranes via in situ formation of Ag nanoparticles has been performed through covalent coating [230] to eliminate the leaching factor that defeats the expected performance of the membranes [231]. In situ synthesis of metal nanoparticles on polymeric surfaces and membranes aided with radiation was also reported elsewhere [232,233,234], whereby the membranes attained an enhancement in the hydrophilicity and antifouling properties. The introduction of a hydrophilic layer onto a membrane surface via thermal grafting [235] and atom transfer radical polymerization [236], followed by loading of nanoparticles, has attracted considerable attention due to the advantage of long-term stability.

**Figure 16:**
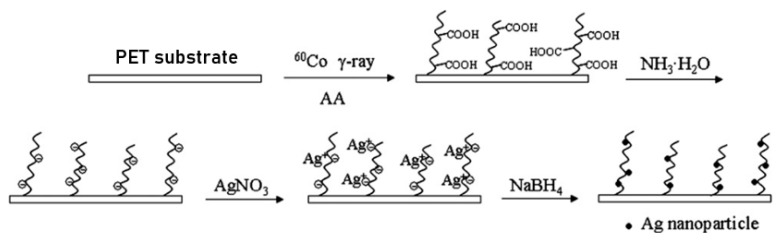
Modification of PET substrate by RIGC of AA using γ-rays with in-situ formation of Ag nanoparticles. Reprinted from [239] with permission from Elsevier.

The metal ions can be imparted to polymer functional groups introduced to the membranes by RIGC via complexation (chelation) from a metal salt solution, which allows non-destructive membrane functionalization. The subsequent reduction in the complexed metal ions leads to the formation of metal nanoparticles covalently bounded to the membrane, adding another significant step towards the development of reactive membranes with biofouling resistance [230]. The reduction reaction of the metal ions in the salt form or metal precursors was carried out either by direct reduction through H radicals that were formed during irradiation [237] or by chemical reduction using a reducing agent [238]. The in situ synthesis of nanoparticles can be performed during or after RIGC. The concept of using RIGC to produce metal nanoparticles is illustrated in Figure 16, whereby, in the early study of Ping et al. [239], Ag nanoparticles were successfully synthesized via chemical reduction of Ag^+^ complexed on the membrane surface by NABH_4_ for improving the antibacterial activity. The grafted PAA acted as stabilizers in the reactions by allowing the retention of Ag nanoparticles between the chains in the graft [237].

In a study by Sawada et al. [238], a PES hollow fiber membrane containing Ag nanoparticles was prepared by RIGC of AAm with UV irradiation in the presence of BP, followed by complexation with AgNO_3_ and subsequent reduction by NABH_4_ [238]. The obtained composite membrane containing Ag nanoparticles in a poly(AAm) gel layer exhibited high potential for applications requiring both organic antifouling and antibacterial properties [238]. Further work by He et al. [240] also reported the loading of Ag nanoparticles on a PES membrane in the same manner, following the UV induced graft copolymerization of the hydrophilic PSBMA, and the membrane showed improved antibacterial properties and biocompatibility.

Another method to load the nanoparticles onto the membranes is by adding pre-synthesized nanoparticles into the monomer solution during the grafting process. El-Arnaouty et al. [207] grafted NIPAM and incorporated ZnO nanoparticles onto PA TFC RO membranes via RIGC, using γ-rays for fouling prevention. The incorporation of NIPAM and ZnO nanoparticles onto PA TFC membranes significantly improved both anti-biofouling and chlorine resistance properties of the commercial PA RO membrane. Other pre-synthesized nanomaterials, such as TiO_2_ nanoparticles [241] and multi-walled carbon nanotubes [242,243], were also introduced to the membranes by RIGC.

## 6. Challenges and Future Directions

The utilization of RIGC for modification of polymeric membranes has some drawbacks. This includes the loss of a part of the mechanical strength of the membranes after modification with RIGC. This is most likely happening when grafting invades the bulk structure of the membranes when high energy radiation is used to initiate graft copolymerization leading to formation of highly stable hydrophilic and amorphous chain grafts within the membrane bulk structure. The mechanical stability is necessary for a membrane to maintain good durability under the flow of the feed solution, especially for high-pressure driven membrane processes such as NF and RO, which use high hydraulic pressures. Therefore, it is very important to optimize the grafting parameters to obtain the desired grafting yields for fulfilling the antifouling properties without compromising the mechanical strength of the membranes. This can be achieved by minimizing the trade-off effects among system parameters such as permeation flux and selectivity in addition to material properties like mechanical strength, and efficient utilization of the hybrid properties imparted from each polymeric component of the grafted membranes.

The use of high energy radiation, such as γ-rays, in modifying membrane surfaces using RIGC with high doses and/or high dose rates is likely to be accompanied by a partial mechanical damage in the grafted membranes. Hence, lower doses and dose rates are preferred to irradiate membranes prior to grafting reactions. However, at a lower dose rate, γ-ray RIGC takes a longer time to achieve a desired grafting yield than EB counterpart. This problem can be solved by using EB with low or medium acceleration energy.

Other concerns about using high energy radiation are the use of conventional solvents to dilute monomers and the need for post-grafting functionalization reactions upon grafting monomers such as acrylates, benzyl chloride, and styrene. This can be resolved by conducting the grafting reaction in an emulsion medium and grafting functional monomers in a single step reaction. Performing grafting in emulsion media not only reduces the monomer consumption and remarkably minimizes the absorbed doses but also helps to maintain the mechanical integrity of the membranes and eventually makes the grafting process more economical and greener.

Apart from that, the increase in the grafting yield usually causes the grafted hydrophilic side chains to induce an excessive swelling, that may promote the dissolution (leaching) of some of the weakly bound grafts carrying functional groups, leading to a decrease in the membrane’s functionality and eventually its performance. Therefore, it is highly important to completely remove the homopolymer that may occlude on the surfaces and pores of the grafted membranes, in addition to controlling the grafting yield.

The use of low energy radiation for modification of membranes also has different set of challenges. Although considered as a simple membrane modifying method, RIGC with UV treatment requires the use of photo-initiators. Besides, the reaction takes a long time to achieve low grafting yield, and the imparted chemical functionalities are only confined to the top surface layers of the membranes. Therefore, this technique is more suitable for applications requiring topical surface modification, rather than the bulk of the membrane. A similar remark can be made for RIGC with plasma, which might also be accompanied by partial degradation in the polymer substrate and changes in the membrane surface morphology induced by plasma etching. In this technique, pores of different sizes are generated at the membrane surface depending on the properties of the applied plasma. Hence, optimization of the plasma treatment parameters is also highly important to achieve desired grafting yields suitable for the target membrane applications.

Most of membrane modifications with RIGC were carried on a laboratory scale level, and there is a need to scale up modification procedures. Several polar monomers were used to improve the membrane hydrophilicity, smoothness, and surface charge, but new modification agents are needed to further improve such changes. For example, zwitterionic compounds perceive more hydration by formation of stronger and more stable electrostatic bonds with water than other hydrophilic compounds and thus, it is recommended to be further explored for membrane modifications by RIGC. It is also recommended to diversify the use of RIGC for the development of dual property membranes such as bipolar membranes, which are very useful for membrane processes driven by concentration and potential gradients. More non-polar polymers should be explored for making membranes suitable for modification by RIGC without compromising their inherent properties.

The utilization of RIGC for membrane modification for applications such as hemodialysis and MBR has its challenges since both applications deal with biological molecules. Hemodialysis membranes are present in the hollow fibers’ configuration having very small dimensions with narrow lumens, which can be easily blocked by the graft chains. The potential of pores’ blocking of the membranes in MBR may induce an affinity for trapping biological molecules causing a reduction in the separation performance. Hence, RIGC parameters such as irradiation time and monomer concentration are of a paramount importance to control the graft propagation reaction and obtain short and highly dense graft chains. This is needed to minimize the graft chains’ entanglements and keep the grafting confined to the desired zone in the membrane (surface) without invading the of pores to prevent their blocking. This further signifies the need to optimize of the grafting parameters during surface modification by RIGC.

The use of radiation sources for in situ formation of metal nanoparticles on the membrane surface by grafting polymer chains of functional monomers acting as anchors to retain the metal nanoparticles on the surface of the membranes after being reduced from their metal complexed forms. As a matter of fact, the presence of metal nanoparticles can also increase the mechanical strength of the membranes. Nevertheless, the RIGC technique for in situ formation of metal nanoparticles is still emerging and there are several challenges related to the nanoparticle loading methods that could hamper the widespread application should be further developed. The introduction of pre-synthesized metal nanoparticles before RIGC would cause a decrease in the available grafting sites due to the aggregation or agglomeration of the nanoparticles, whereas the incorporation of nanoparticles after grafting is dependent on the reduction reaction parameters and the stability of the formed nanoparticles. Typically, the use of conventional reducing agents needs to be minimized and replaced with alternative green reducing agents, if possible, to keep the reaction greener. This should be accompanied by optimization of the complexed metal reduction parameters to control the size and the shape of the formed nanoparticles. 

Silver nanoparticles are the most used nanometals for membrane modification so far, and hence other metals like copper and metal oxides such as TiO_2_ or ZnO should be explored. Since membrane modification involves an additional cost, more efforts are needed to reduce the overall antifouling membranes’ fabrication cost and test the modified membranes for in situ treatments of real samples of saline or wastewater and widen their applications.

## 7. Conclusions

The developments in the modification of polymeric membranes using RIGC with high- and low-energy radiation sources for fouling prevention were reviewed, with special attention given to solid/liquid separation systems such as pressure driven membrane systems. The use of this eco-friendly method, with less monomer consumption and minimal residue production for membrane modification with plasma, UV, γ-rays, and EB treatments, has been intensified in the past two decades. The various techniques adopted to impart surface chemical functionalities through different strategies using RIGC were found not only to be effective and feasible in conferring desired properties for reducing the organic membrane fouling/biofouling, but also to improve the membrane separation’s performance and durability. The selection of RIGC methods depends on the type of the membrane application and the desired level of modification. In this regard, the use of high energy radiation (γ-rays and EB) for grafting initiation is desired to achieve controlled grafting, varying from the surface to bulk of the membranes, unlike low energy radiation (UV and plasma), which only allow functionalized grafts to be only confined to surface top layers. RIGC technique was also found to be most suitable for in situ formation of metal nanoparticles on the surface of the membranes, to confer antimicrobial properties and enhance the mechanical stability by having nanocomposite structures. The membranes modified by RIGC were utilized in various pressure driven processes involving water/wastewater treatments and showed strong potentials for other applications such as hemodialysis, ED, and RED. Finally, more work is needed to overcome the critical challenges limiting the widespread applications of the membrane modification with RIGC for industrial scale applications. Particularly, attention should be given to application of EB as the most suitable radiation source for the development of a roll-to-roll process for production of polymeric membranes with antifouling properties. Moreover, cheap monomers should be used, and the reaction should be preferably conducted in an emulsion medium to make the process greener and economic. This should be accompanied by optimization of the irradiation and grafting reaction parameters to achieve desired levels of surface modifications. More collaborations between researchers and industrial practitioners are also highly sought to accelerate the deployment of the technology. If it hits the right notes, with extra support from policymakers and active participation from key players in research centers and industries, RIGC could be a highly demanded modification technique to serve industrial needs for elimination of fouling from membranes.

## Figures and Tables

**Table 1:** Fouling profiles of different membrane processes.

Process	Membrane Type	Driving Force	Feed	Class of Fouling	Foulant	Severity
MF	Asymmetric microporous, 0.1 to 10 µm	TMP 0.2–5 bar	Wastewater	Biofouling, colloidal fouling	Suspended solids and bacteria	Medium
UF	Asymmetric microporous, 0.01 to 0.1 µm	TMP 1–10 bar	Water and wastewater	Biofouling, colloidal fouling	Proteins and pigments	Medium
NF	Thin film composite (TFC), 1 to 10 nm	TMP 5–10 bar	Brackish water	Organic fouling, inorganic scaling	Pigments, divalent ions, glucose, and lactose	High
RO	TFC, 0.1 to 1 nm	TMP 10–50 bar	Seawater	Colloidal fouling, inorganic scaling	Dissolved salts and monovalent ions	High
ED/EDR	CEM and AEM	Electrical potential gradient	Brackish water	Inorganic scaling	Inorganic colloids and insoluble salts	Low
FO	Asymmetric skin-type, <0.001 µm	Chemical potential gradient	Wastewater	Organic fouling, colloidal fouling	Micropollutants and salt	Medium
PRO	Asymmetric skin-type, <0.001 µm	Chemical potential gradient	Wastewater	Organic fouling, colloidal fouling	Micropollutants and salt	Medium
RED	CEM and AEM	Electrical potential gradient	Seawater and river water	Inorganic scaling	Inorganic colloids and divalent ions	Low
Dialysis	Asymmetric microporous, 0.01 to 0.1 µm	Concentration gradient	Blood	Biofouling	Proteins, blood cells and platelets	Medium

**Table 2:** Summary of merits and demerits of common polymeric membranes modification methods. Adapted from [122].

Modification Method		Merits	Demerits	Remarks
Physical method	Dip coating	Simple and flexible technique to optimize hydrophilicity, smoothness, and surface charge of the membrane surface	Physical (non-covalent) coating is easily worn out and detached from polymer substrate. Non-uniform coating across the polymer substrate	This method is very outdated and irrelevant with current advance in technology. Results are inconsistent
	Layer by layer assembly	Film thickness can be controlled at the nanometers scale. Deposited layer can be optimized	Deposited layers may vary in thickness. Hard to control uniformity of each layer	Results are inconsistent
	Blending	Easiest and simplest method. Very straightforward and does not involve chemical reaction. Addition of inorganic particles enhance mechanical strength	Heterogeneous distribution of inorganic particles. High tendency for leaching of particles from membrane and particle agglomeration within polymer matrix	This method is very outdated and irrelevant with current advance in technology. Results are inconsistent
Chemical method	Interfacial polymerization	Well-established method to prepare RO and NF membranes with 99% salt rejection. Able to form a very thin PA film on top of polymer substrates	Difficult to control uniformity of the film across the polymer substrate. Trade-off between permeability and rejection	The thin film should be embedded with functional nanomaterials, forming thin film nanocomposite to solve the trade-off issue
	Chemical induced graft copolymerization	Cheap chemical initiators and effective in achieving significant grafting yields	Leaves residues, causing environmental pollution. Difficult to control grafting yield	The use of green solvent with minimal volume should be considered
	RIGC using plasma treatment	Simple process without any pollution to modify the polymeric surfaces without altering their bulk properties, allowing functionalization with ionic group for hosting biocides	The range of chemical groups available for surface modification is limited, posing a challenge to effectiveness for deterring bacterial adhesion. Not suitable for large scale applications	More suitable for biomedical application that requires limited surface modification, such as catheters and cannulas, in addition to bio-medical coatings to various surfaces
	RIGC using UV treatment	Simple, inexpensive and can easily modify polymer surfaces	It yields low grafting level, which is confined to surface, takes long treatment time, and requires the use of photo-initiator. Not suitable for large scale applications	More suitable for surface modification that can help improving wettability and resistance to bacterial colonization and biofilm formation
	RIGC using γ-rays	Simple but slower that EB, allows bulk grafting depending on absorbed dose and dose rate. Widely applied and most suitable for simultaneous grafting in bulk solution	Grafting takes longer than EB. The Co60 source continues to decay and thus dose rate reduces steadily. Requires adjustment of reaction parameters	Green grafting reactions can be conducted in emulsion to significantly reduce monomer consumption and absorbed dose and improve the process economy
	RIGC using EB	Simple and very fast. Allows surface as well as bulk grafting depending on acceleration energy. Leaves no detrimental residues. Can be initiated with EBs with wide range of energies	High cost of infrastructure for irradiation. Grafted materials are likely to sustain mechanical damage when high doses and dose rates are used	More convenient for practical applications and is more suitable for scale up and development of semi-continuous lines for industrial applications

**Table 3:** Summary of previous studies on application of RIGC using plasma treatment for fouling prevention.

Substrate	Grafted Monomer	Main Finding(s)	Refs
CTA	AA	Art was more effective than CO_2_ to increase water flux and decrease reverse salt flux and fouling tendency	[141]
PE	AAm	Membranes of different functional groups with opposite surface charges can be utilized for covalent immobilization of protein	[138]
PE	AA	The modified membrane showed a significant increase in hydrophilicity, water flux, and BSA solution flux	[139]
PES	PEG, amines, zwitterionic compounds	High stability of PEG polymer chain and protein-resistance of PEG grafted PES were achieved compared to using UV as radiation source	[123]
PES	AAm	Due to the improved surface hydrophilicity, the grafted membrane was less susceptible to BSA protein adsorption and had higher flux recoveries after cleaning	[124]
PES	NVP	BSA fouling was significantly reduced, and the cleaning of modified membranes was easier to recover permeation flux	[125]
PES	AA	Modified membranes were hydrophilic, less prone to protein fouling, and had a higher pure water flux	[126]
PES	AA	The grafting of AA occurred on the membrane surface and on the pore walls inside the membranes, which enhanced the fluxes and the antifouling properties of the membranes	[127]
PES	SPMA	Outstanding water–oil flux by the modified membranes was achieved at grafting temperature of 65 °C and grafting yield of 0.489 mg/cm^2^, followed by flux recovery of 87.5%	[128]
PES	AA, HEMA	The membrane modified with HEMA has reduced fouling propensity due to the absence of deep pockets in its structure, whereas PES-*g*-PAA had a damaged membrane structure	[129]
PS	AA	Grafting in solution resulted in hydrophobic membranes with significantly smaller pore sizes. When grafting in the vapor phase, AA grafted surface layer closely resembled pure PAA, which was hydrophilic in a basic environment	[135]
PS	DMAEMA, AA	The adsorption of lysozyme on the DMAEMA grafted membrane was greatly reduced. AA grafted membrane, which exhibited a stronger negative surface charge, has caused reduction in BSA adsorption due to the increased electrostatic repulsive force	[136]
PS, PAN	HEMA, AA, MAA	After grafting with HEMA, the water contact angles of PAN and PSf reduced. These membranes also had significantly less fouling and better protein UF performance	[134]
PAN	NVP	With an increase in both graft reaction time and grafting medium temperature, water flux decreased significantly	[142]
PPO	SSS	Micellar-enhanced UF of mixtures of the 2,4-D herbicide and hexadecyltrimethylammonium bromide was much better with bipolar amphoteric membranes with combined sulfonic acid and allylamine	[144]
PP	EGDME	Polyethylene oxide-like PP with antiplatelet behavior was formed using EGDME as monomer, with almost no sign of platelet adhesion and accumulation	[147]
PP	NVP	Flux recovery, flux reduction, and relative flux ratio were 53% higher, 17.9% lower, and 79% higher, respectively, than the neat PP membrane	[143]
PA TFC	MAA	The optimal membrane surface’s onset time for gypsum scaling was delayed by a factor of 2–5, hence reduced the propensity for mineral scaling	[45]
PA, polyester	PEG	The PA-*g*-PEG and polyester-*g*-PEG membranes had similar hydrophilicity, and they showed 96% reduction in biofouling caused by *Listeria monocytogenes*	[155]
PVDF	AA	Due to the presence of PVDF-*g*-PAA, less irreversible fouling was detected in the modified PVDF membrane	[130]
PVDF	GMA-IDA	Surface hydrophilicity of the PVDF-GMA-ID bipolar membrane was increased	[131]
PVDF	TMA, SPMA	In static conditions, BSA and lysozyme adsorption tests, as well as an *Escherichia coli* attachment test, revealed the reduced biofouling by pseudo-zwitterionic PVDF membranes	[132]
PVDF	AA and DMAEA	The contact angle of bipolar membranes decreased	[133]
ePTFE	AA	The grafting of AA onto ePTFE resulted in highly hydrophilic membranes with high water uptake	[140]
ePTFE	PEGMA	The surface hydrophilicity of PEGMA grafted ePTFE membranes increased, which reduced protein adsorption and platelet adhesion	[154]
ePTFE	PSBMA, PEGMA	The zwitterionic PSBMA grafted ePTFE membrane had the best non-bio adhesive character against biomacromolecules and cells	[158]

**Table 4:** Summary of previous studies on application of RIGC using UV treatment for fouling prevention.

Substrate	Grafted Monomer	Main Finding(s)	Ref.
PEEK	HEA	Due to the increased surface hydrophilicity, the irreversible fouling of optimized membrane decreased from 51% to 10.9%	[99]
PEEK	MPC	The membrane displayed high wettability and high anti-protein adsorption	[173]
PVDF	AA, HEMA, PDA, EDA	Antifouling properties such as flux recovery and fouling resistance of modified membranes were improved	[180]
PVDF/PES	NVP	Grafted membrane showed good fouling resistance due to the decreased BSA adsorption, reduced fouling degree by 66%, and better flux recovery by 32% after chemical cleaning	[192]
PA TFC	NIPAM	Change in surface chemistry of grafted membrane due to formation of temperature responsive poly(NIPAM) hydrogel improved fouling resistance and salt rejection	[189]
PES	NVP, HEMA, AA, AAG, SPMA, AMPS	NVP, AMPS, and AA-modified membranes had high protein retention, high solution flux, and low irreversible fouling	[174,178]
PES	AA, HEMA, PDA, EDA	The membranes suffered a decrease in permeation of pure water and milk water but with improved protein rejection. PES membrane grafted with poly(HEMA) had the best antifouling properties	[181]
PES	PEG, NVF, NVA, MVA	PES membrane grafted with PEG–NVA binary monomer pair displayed the best BSA fouling resistance	[183]
PES	AA	Modified MF membranes had lower permeability but showed 100% flux recovery after cleaning, following the filtration of *Escherichia coli*	[175]
PES	AA	Modified NF membranes exhibited higher flux, higher humic acid rejection, and lower irreversible fouling	[176]
PES	AA, AAm	The separation ability and flux recovery ratio of PES-*g*-AAm surpassed PES-*g*-AA and unmodified PES membranes	[177]
PES	PEGMA	Modified membrane with high monomer concentrations (40 g/L) and medium irradiation times (1.5–3 min) demonstrated greater flux, fouling resistance, and higher protein rejection	[196]
PES	PEGMA, MMESPAB	PEGMA and MMESPAB grafted PES membranes displayed far better adsorptive fouling resistance than unmodified PES membrane	[186]
PES	NVP, NVF, NVC	In comparison to the initial membrane, modified membranes showed higher fluxes and less BSA fouling, especially for PES-*g*-poly(NVP) membrane	[159]
PES	NVP	Membranes irradiated for 60 s had a lower fouling tendency. However, under long irradiation times, the pore structure increased in size, increasing membrane fouling	[162]
PES	NVP	Both the dip and immersion modification techniques produced membranes with increased wettability and reduced irreversible adsorptive fouling	[160]
PES	NVP, 2-mercaptoethanol	The permeability of the membranes decreased as the grafting yield increased	[161]
PES	Poly(CBOH)	The membrane had a switchable feature between the anti-fouling, zwitterion mode and an anti-microbial, quaternary amine mode by adjusting the pH	[188]
PES, PVDF	AMPS, qDMAEMA, HEMA	HEMA was less susceptible to fouling on the neutral hydrophilic membrane surface than on the charged membranes	[172]
PES, PVDF	qDMAEMA, AMPS	Modified membranes were more biofouling resistant. The number of proliferated bacterial cells from countable colonies was much lower for qDMAEMA grafted membranes	[170,171]
PS	MA	Hydrophilicity of graft copolymer membrane increased as the MA grafting yield increased. The antifouling property of the membrane was improved	[184]
PS	BHMBA	Modified membranes had low surface roughness which corresponded to the improved antibiofouling property and excellent antibacterial properties against *Escherichia coli*	[198]
PS	MPDSAH	As the grafting yield increased, the modified membranes’ hydrophilicity and antifouling properties improved	[185]
PAN	AA, HEMA, PEGMA	Adsorption and fouling were reduced for both negatively and positively charged membranes	[179]
PP	AA, AAm	The modified membranes performed better in the MBR than the unmodified ones, with the AA grafted membrane having the best antifouling properties	[168]
PP	GAMA	After 70 h of continuous operation in the MBR, the modified membranes had reduced water flux of up to 87.2%, at increased length of the grafted chains	[165]
PP	HEMA	Because of the increased surface hydrophilicity, the modified membrane demonstrated improved protein resistance and hemocompatibility	[166]
PP	AAm	The inner part of the membrane had a higher grafting yield than the outer part. The modified membrane had better flux recovery of approximately 70%	[167]
PP	NVP	The surface hydrophilicity increased with the increase in grafting yield. The amounts of adsorbed BSA and adhered platelets on membrane decreased substantially	[163]
PP	NVP	The membrane with iodine complex has a desirable antibacterial property against *Escherichia coli*, *Staphylococcus aureus*, and *Candida albicans*	[164]
PE	PDMS, PEG	The membrane showed reduced fouling towards *Pseudomonas aeruginosa* as the membrane surface became smoother and more hydrophilic, with decreased membrane charge	[169]
PE	MPC, NVP, AAm, MPEG	The poly(MPC) and polyvinylpyrrolidone (PVP) grafts on PE membrane substantially helped to reduce the plasma protein adsorption and the platelet adhesion	[164]
PI	AA, HEMA, PDA	Pure water and milk water permeation of PI membranes decreased while the protein and salt rejection increased after grafting, especially with PDA monomer	[182]
PET	PZC	The effectiveness of PZC in preventing membrane blockage and filtering platelets from blood plasma helped the grafted membrane to exhibit superior anti-biofouling properties	[187]

**Table 5:** Summary of previous studies on application of RIGC using γ-rays for fouling prevention.

Substrate	Grafted Monomer	Main Finding(s)	Ref.
PES	SSS, AA, NVP	All modified membranes’ contact angles, protein adsorption, and platelet adhesion decreased. The modified membranes had good hemocompatibility	[206]
PES	MAA	Membrane prepared from PES-*g*-poly(MAA) powder exhibited the flux of acid solution up to four times that of basic solution	[213]
PVDF	HEA	The grafted membrane had lower pure water flux than the control membrane but showed noticeably higher BSA solution flux than the pure water flux	[212,215]
PVDF	NVP	As the grafting yield increased, the contact angle decreased, and water uptake, RMS, water flux, pore size, and water flux recovery of the membrane increased	[203]
PVDF	PVA	The modified membrane achieved oil rejection up to 99.5%. The oil fouling on modified PVDF membranes was almost reversible, with flux recovery of 98%	[211]
PVDF	NVP	Maximum grafting yield of 17.7% was obtained when reaction was carried out in water for 3 h at a monomer concentration of 20% (*v*/*v*) and an absorbed dose of 40 kGy	[208]
PVDF	NIPAM	The increased amount of PVDF-*g*-poly(NIPAM) in membrane enhanced its hydrophilicity and heightened the water flux	[204]
PP	HEMA	With increased grafting yield, the modified membranes’ contact angle decreased. The modified membrane had a higher solution flux, lower BSA adsorption, and better flux recovery	[209]
PP	MMA	Maximum grafting yield of 85% was obtained at 25 kGy radiation dose, 0.04 wt% inhibitor concentration, 6 wt% monomer concentration, 60 °C reaction temperature, and 120 min reaction time	[205]
PP	NVP	The amounts of adsorbed BSA and adhered platelets on membrane decreased substantially	[163]
PP	NVP	The increased roughness of the grafted membrane surface was due to the formation of grafted chains on the polymer surface	[214]
PA TFC	PVA	The surface hydrophilicity of the PVA grafted RO membrane was significantly increased and the membrane had excellent antifouling property	[210]

**Table 6:** Summary of previous studies on application of RIGC using EB for fouling prevention.

Substrate	Grafted Monomer	Main Finding(s)	Ref.
PES	Carboxylic, sulfonic and phosphoric acids, amines, alcohols, zwitterionic compounds	This modification resulted in significantly reduced protein adsorption at the membrane surface with selected functional molecules	[222]
PS	AMPS	The grafted membranes achieved NF performance at a relatively large pore size due to the high negative charge density resulting from the high grafting yield	[223]
PTFE	AA, SSS	AA/SSS binary monomers had a synergistic effect on grafting yield and membrane hydrophilicity with increase in AA content and irradiation dose	[219]
PVDF	AA, SSS	The surface hydrophilicity of the grafted membrane improved significantly	[218]
PVDF	PEGMA	Immobilizing hydrophilic comb-like poly(PEGMA) brushes on the PVDF membrane surface enhanced both hydrophilicity and fouling resistance	[220]
PVDF	PEG, PLU, PVA, PVP, PAH, PSS	Improved membrane wettability was indicated by lower water contact angles. Hemocompatibility tests revealed no unwanted hemolysis, and hydrophilic polymers were found to reduce blood coagulation	[224]
PVDF	Trypsin	The modified membrane had significantly improved antifouling properties. The fouling layer formed on the membrane’s surface can be actively degraded during filtration, restoring the membrane’s original permeability	[217]
PVDF/PVP	L-cysteine, phosphocholine, DMAEMA	Membranes exposed to absorbed dose of 10 kGy had higher permeate flux and lower cake resistance. The membrane irradiated with 10 kGy in the presence of L-cysteine had the best long-term antifouling capacity	[225]
PVDF-*co*-HFP	NVP	The PVP grafts on the membrane was capable of hosting I_2_, thus imparting a very strong antimicrobial activity to the membrane, which further lessened the biofouling	[226]
PVDF	GMA, EDMA	Addition of EDMA only resulted in a denser membrane structure and reduced the amount of oxirane groups converted to sulfonic groups. The PVDF-*g*-poly(GMA) membrane had a higher ion exchange capacity and improved hydrophilicity with electrostatic effect	[25]

## Data Availability

Data are contained within the article.

## Abbreviations

AA	Acrylic acid
AAG	2-acrylamidoglycolic acid
AAm	Acrylamide
AEM	Anion exchange membrane
AFM	Atomic force microscopy
AMPS	2-acrylamido-2-methyl-1-propanesulfonic acid
APP	Atmospheric pressure plasma
BHMBA	N-(3-tert-butyl-2-hydroxy-5-methylbenzyl) acrylamide
BP	Benzophenone
BSA	Bovine serum albumin
CEM	Cation exchange membrane
CTA	Cellulose triacetate
DMAEA	2-(dimethylamino) ethyl acrylate
DMAEMA	2-(dimethylamino) ethyl methacrylate
EB	Electron beam
ED	Electrodialysis
EDA	Ethylene diamine
EDMA	Ethylene glycol dimethacrylate
EDR	Electrodialysis reversal
EGDME	Ethylene glycol dimethyl ether
ePTFE	Expanded polytetrafluoroethylene
FO	Forward osmosis
GAMA	D-gluconamidoethyl methacrylate
GMA	Glycidyl methacrylate
GMA-IDA	Glycidyl methacrylate-iminodiacetic acid
HEA	Hydroxyethyl acrylate
HEMA	2-hydroxyethyl methacrylate
HFP	Hexafluoropolypropylene
LPP	Low pressure plasma
MA	Methyl acrylate
MAA	Methacrylic acid
MBR	Membrane bioreactor
MF	Microfiltration
MMA	Methyl methacrylate
MMESPAB	N,N-dimethyl-N-(2-methacryloyloxyethyl-N-(3-sulfopropyl)ammonium betaine
MPC	2-methacryloyloxyethyl phosphorylcholine
MPDSAH	[3-(methacryloylamino)propyl]-dimethyl (3-sulfopropyl) ammonium hydroxide
MPEG	Methacryloyl poly(ethylene glycol)
MVA	N-methyl-N-vinylacetamide
NF	Nanofiltration
NIPAM	N-isopropyl acrylamide
NVA	N-vinylacetamide
NVC	N-vinyl-caprolactam
NVF	N-vinyl formamide
NVP	N-pyrrolidone
PA	Polyamide
PAH	Polyallylamine hydrochloride
PAN	Polyacrylonitrile
PDA	2,4-phenylenediamine
PDMS	Poly(dimethylsiloxane)
PE	Polyethylene
PEEK	Poly (ether ether ketone)
PEG	Polyethylene glycol
PEGMA	Poly(ethylene glycol) methacrylate
PES	Polyethersulfone
PET	Polyethylene terephthalate
PI	Polyimide
PLU	Pluronic
PP	Polypropylene
PPO	Poly(phenylene oxide)
PRO	Pressure retarded osmosis
PS	Polysulfone
PSBMA	Poly(sulfobetaine methacrylate)
PSS	Polystyrene sulfonate
PTFE	Polytetrafluoroethylene
PVA	Polyvinyl alcohol
PVDF	Polyvinylidene fluoride
PVP	Polyvinylpyrrolidone
PZC	Pseudo-zwitterionic copolymer
qDMAEMA	Quaternized 2-(dimethylamino) ethyl methacrylate
RED	Reverse electrodialysis
RIGC	Radiation induced graft copolymerization
RMS	Root-mean square
RO	Reverse osmosis
SPMA	3-sulfopropyl methacrylate
SSS	Sodium styrene sulfonate
TFC	Thin film composite
TMA	Trimethylammonium
TMP	Transmembrane pressure
UF	Ultrafiltration
UV	Ultraviolet
